# Spatial selectivity of ATase inhibition in mouse models of Charcot–Marie–Tooth disease

**DOI:** 10.1093/braincomms/fcae232

**Published:** 2024-07-09

**Authors:** Gonzalo Fernandez-Fuente, Mark A Farrugia, Yajing Peng, Andrew Schneider, John Svaren, Luigi Puglielli

**Affiliations:** Department of Medicine, School of Medicine and Public Health, University of Wisconsin-Madison, Madison, WI 53705, USA; Waisman Center, University of Wisconsin-Madison, Madison, WI 53705, USA; Department of Medicine, School of Medicine and Public Health, University of Wisconsin-Madison, Madison, WI 53705, USA; Waisman Center, University of Wisconsin-Madison, Madison, WI 53705, USA; Department of Medicine, School of Medicine and Public Health, University of Wisconsin-Madison, Madison, WI 53705, USA; Waisman Center, University of Wisconsin-Madison, Madison, WI 53705, USA; Waisman Center, University of Wisconsin-Madison, Madison, WI 53705, USA; Waisman Center, University of Wisconsin-Madison, Madison, WI 53705, USA; Department of Comparative Biosciences, School of Veterinary Medicine, University of Wisconsin-Madison, Madison, WI 53706, USA; Department of Medicine, School of Medicine and Public Health, University of Wisconsin-Madison, Madison, WI 53705, USA; Waisman Center, University of Wisconsin-Madison, Madison, WI 53705, USA; Geriatric Research Education Clinical Center, Veterans Affairs Medical Center, Madison, WI 53705, USA

**Keywords:** ATase, acetylation, proteostasis, endoplasmic reticulum, Charcot–Marie–Tooth disease

## Abstract

The endoplasmic reticulum acetylation machinery has emerged as a new branch of the larger endoplasmic reticulum quality control system. It regulates the selection of correctly folded polypeptides as well as reticulophagy-mediated removal of toxic protein aggregates with the former being a particularly important aspect of the proteostatic functions of endoplasmic reticulum acetylation. Essential to this function is the Nε-lysine acetyltransferase activity of acetyltransferase 1 and acetyltransferase 2, which regulates the induction of endoplasmic reticulum–specific autophagy through the acetylation of the autophagy-related protein 9A. Here, we used three mouse models of Charcot–Marie–Tooth disease, peripheral myelin protein 22/Tr-J, C3-peripheral myelin protein 22 and myelin protein zero/ttrr, to study spatial and translational selectivity of endoplasmic reticulum acetyltransferase inhibitors. The results show that inhibition of the endoplasmic reticulum acetyltransferases selectively targets misfolding/pro-aggregating events occurring in the lumen of the organelle. Therefore, they establish acetyltransferase 1 and acetyltransferase 2 as the first proven targets for disease-causing proteotoxic states that initiate within the lumen of the endoplasmic reticulum/secretory pathway.

## Teaser

Here, Fernandez-Fuente *et al*. used three mouse models of Charcot–Marie–Tooth disease, to study spatial and translational selectivity of acetyltransferases inhibitors. The results establish the acetyltransferases as the first proven targets for disease-causing proteotoxic states that initiate within the lumen of the endoplasmic reticulum/secretory pathway.

## Graphical Abstract

**Graphical Abstract:**
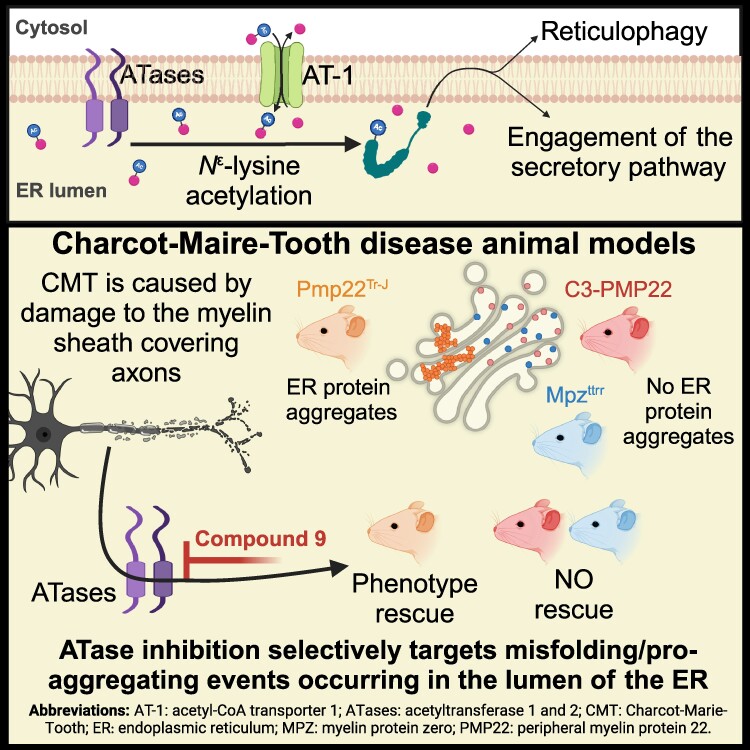

## Introduction

A fundamental task of the endoplasmic reticulum (ER) is to make proteins that can then engage the secretory pathway to reach their final destination within the cell or be secreted to the extracellular *milieu*. Quality control mechanisms are in place to ensure that only correctly folded polypeptides can leave the ER. Quality control mechanisms are also in place to remove misfolded/unfolded polypeptides that would otherwise accumulate in the ER and cause proteotoxicity.^1,2^ By ensuring the continuous and efficient removal of toxic protein aggregates, the ER-specific autophagy (alternatively referred to as reticulophagy, ER-phagy or ER-associated degradation type II) represents a fundamental component of the ER quality control system. Defective removal of toxic protein aggregates through autophagy has been linked to different diseases across lifespan.^3-6^ As such, it is not surprising that improving normal proteostatic mechanisms represents an active target for biomedical research.

The ER acetylation machinery has emerged as a novel branch of the larger ER quality control system. It regulates the selection of correctly folded polypeptides, but also reticulophagy-mediated removal of toxic protein aggregates.^7-16^ ER acetylation is ensured by AT-1/SLC33A1, a membrane transporter that allows entry of cytosolic acetyl-Coenzyme A (acetyl-CoA) into the ER lumen, and ATase1/NAT8B and ATase2/NAT8, two ER-based acetyl-CoA:lysine acetyltransferases that use acetyl-CoA to acetylate ER cargo proteins within the ER lumen following their initial tertiary-state folding.^7,16-18^

The disposal of toxic protein aggregates through reticulophagy is an important aspect of the proteostatic functions of ER acetylation. This process requires ATase1- and ATase2-mediated acetylation of the ER-bound autophagy protein ATG9A, which in turn regulates the recruitment of the autophagy core machinery.^13,19^ In the mouse, reduced ER acetylation causes increased induction of reticulophagy, while increased ER acetylation has the opposite effect.^9,11,12^ Genetic disruption or biochemical inhibition of the ATases results in activation of reticulophagy, as well as rescue of disease-associated proteotoxicity.^10,12,14,15,20^ Disease-causing mutations in genes involved in the regulation of the proteostatic functions of the ER acetylation machinery (i.e. *AT-1/SLC33A1*, *FAM134B* and *HSPB1*) have been associated with different forms of hereditary sensory and autonomic neuropathy/hereditary motor neuropathy (HSAN/HMN) and spastic paraplegias (SPGs).^21-26^ Finally, HSAN/HMN-causing mutations have been associated with mislocalization of ATG9A and impaired autophagic degradation of pathogenic aggregates.^27,28^

In terms of translational output for autophagy-based strategies, a fundamental need is to selectively target autophagy to a specific cellular location. Studies conducted in different mouse models of proteotoxicity indicate that increasing reticulophagy through targeted inhibition of the ATases is a valid strategy to rescue disease-causing proteotoxic states of the ER and secretory pathway.^10,12,14,15,20^ However, these studies were not designed to discriminate between disease-causing events that force a target protein to misfold/aggregate (i.e. a mutation) and disease-causing events that do not (i.e. a gene duplication) or between misfolding events that affect the luminal versus the cytosolic portion of ER-bound membrane proteins. This is a particularly important aspect of ‘personalized medicine’ since different genetic events (i.e. mutation, duplication or deletion) can be the underlying pathogenic mechanism. Sometimes, these different genetic events are associated with the same group of diseases (i.e. HSAN/HMN/SPGs) or even target the same protein (i.e. peripheral myelin protein 22, PMP22).^29,30^

Here, we used three mouse models of Charcot–Marie–Tooth disease, a peripheral form of neuropathy, to target the above limitations. The effectiveness of targeting ATase activity was tested using neuropathy models that involve duplication or misfolding mutations of two abundant peripheral myelin proteins in Schwann cells of peripheral nerves. The results show that ATase inhibition selectively targets misfolding/pro-aggregating events occurring in the lumen of the ER, thus establishing the ATases as the first proven targets for proteotoxic states that initiate within the lumen of the ER.

## Materials and methods

### Animals

All the animals used in this study were obtained from the Jackson Laboratory. Strains B6.Cg-Tg(PMP22)C3Fbas/J (#JAX:030052), B6.D2-*Pmp22^Tr−J^*/J (#JAX:002504) and B6.Cg-*Mpz^ttrr^*/GrsrJ (#JAX:010494) were maintained on the C57BL/6J (#JAX:000664) genetic background. B6.Cg-Tg(PMP22)C3Fbas/J and B6.D2-*Pmp22^Tr−J^*/J mice were bred as heterozygous while B6.Cg-*Mpz^ttrr^*/GrsrJ were bred as homozygous.

Mice were housed and fed as described.^31,32^ The diet with Compound 9 (C9; 1 mg/g) was manufactured by Bio-Serv.^12,15,20^ Treatment with C9 began at weaning and continued throughout the entire life of the animals. All animal experiments were approved by the Institutional Animal Care and Use Committee of the University of Wisconsin-Madison (protocol #M005120) and performed in accordance with the National Institute of Health Guide for the Care and Use of Laboratory Animals. Wild-type (WT) littermates were used as controls throughout the study. The age and sex of the animals at time of experimentation are specified in the figure and figure legends. Genotyping from tail DNA was performed at weaning by Transnetyx using real-time polymerase chain reaction.

### Behaviour testing

All behavioural assays were conducted at the Waisman Center Behavioral Testing Service (Madison, WI, USA). All mice received a minimum of 30 min acclimation time to the testing room prior to each behaviour assay.

#### Phenotypic severity score

We used a simple composite phenotype scoring system for evaluating mouse models of cerebellar ataxia described previously.^33^ Blind analysis of the animals was performed for three different tests: ledge, hindlimb clasping and gait. Individual measures are scored on a scale of 0 to 3, with 0 representing an absence of the relevant phenotype and 3 representing the most severe manifestation (Supplementary Table 1).

#### Open field exploration

The specified protocol was performed according to Rigby *et al*.^31,32^ Data were recorded using the Omnitech Fusion system.

#### Hot plate

Mice were placed on a hot plate (Columbus Instruments, hot plate analgesia metre) to evaluate the reaction time. The reaction time was scored when the animal jumped or licked its paws. A cut-off of 40 s was used to avoid any paw damage. Five reaction times were determined for each mouse with a latency of at least 15 min apart between measurements. The results shown for each animal are the average of the middle three values.

#### Grip strength

Grip strength was measured using an Ametek Chatillon DFE II (Columbus Instruments, grip strength metre). The mouse was held by the base of the tail above a wire bar connected to the force gauge. The mouse was placed in a position that allowed it to grasp the wire with its forepaws and then pulled away from the bar at a constant speed. The maximum force generated just before the mouse lost its grasp was recorded. This was repeated five times for each animal. The results shown for each animal are the average of the middle three values.

#### Inverted screen

Mice were removed from the home cage and placed on top of the screen. A gentle shake of the screen was performed to make sure the mouse had gripped the screen. Then, the screen was carefully inverted at 30 cm over the empty cage so that the mouse was upside down on the screen. Time was measured from the inversion moment to record the time latency to fall. This was repeated five times for each animal with a latency of at least 10 min between measurements. The results shown for each animal are the average of the middle three values.

#### Balance beam

The balance beam apparatus is composed of one smooth, plastic beam 70 cm in length and 3 cm in diameter. The beam is securely suspended 25 cm above the surface. Enclosed safe house is placed at the escape end of the beam, and bedding is added to encourage the mouse to enter. A training session was conducted the day before the test session. The test session consists of five runs per animal, and the results are shown as total successful runs per animal (0–5 score).

### Electron microscopy

Following CO_2_ euthanasia, sciatic nerves were extracted and fixed in 2.5% glutaraldehyde in 0.1 M phosphate buffer overnight at 4°C. The specified protocol involving fixation, dehydration, embedding and sectioning was performed according to Peng *et al*. and Pehar *et al*.^9,34^ The sectioned samples were viewed at 80 kV on a Philips CM120 transmission electron microscope equipped with AMT BioSprint12 digital camera (AMT Imaging Systems).

### Morphometry

Non-overlapping electron micrographs of sciatic nerves were analysed for axon diameter and g-ratio. A minimum of 100 randomly selected fibers were analysed per animal using the g-ratio plug-in of the ImageJ software, which allowed for semi-automated analysis of randomly selected sets of fibres.^35^ For each nerve, the percentage of non-myelinated axons was calculated by direct count from the electron microscopy micrographs.

### Protein extraction and western blotting

Detergent-soluble and detergent-insoluble fractions were generated as described.^10^ The following primary antibodies were used in this study: anti-DDK #TA50011-100 antibody from OriGene and anti-HAtag #26183 antibody from Invitrogen. Goat anti-mouse IRDye 800CW and 680RD-conjugated secondary antibodies (LI-COR Biosciences, #926-32210, #926-68070) were used for infra-red imaging (LI-COR Odyssey Infrared Imaging System; LI-COR Biosciences).

### Cell culture and plasmids

CHO-K1 (Ovary Chinese Hamster, ATCC CCL-61^™^) cells and mouse embryo fibroblasts were grown in Dulbecco’s modified Eagle’s medium (DMEM; Corning #10-017-CV) supplemented with 10% foetal bovine serum (Corning #35-010-CV) and 1% penicillin/streptomycin/glutamine (Gibco #10378016). Cells were maintained at 37°C in a humidified atmosphere with 5% CO_2_. Cells were transiently transfected with Lipofectamine 2000 (Invitrogen #11668019) using the following constructs: mCherry-ER-3 plasmid, a gift from Michael Davidson (Addgene plasmid #55041); PMP22 human-tagged ORF clone (OriGene #RC216500); and MPZ human-tagged ORF clone (OriGene #RC202450). Additionally, plasmids for human PMP22_Tr-J_ (T47C, Leu to Pro) and human MPZ_ttrr_ (GTGC deletion and TGTATGCAATGC duplication) were developed in our laboratory using site-directed mutagenesis (New England Biolabs Q5 Site-Directed Mutagenesis Kit #E0554S). Primers for Tr-J mutation are as follows: 5′-GTCGCGGTGCCGGTGCTGCTG-3′ and 5′-GTGGAGGACGATGATACTCAGCAAC-3′. Primers for MPZ fragment deletion are as follows: 5′-TGTATGCAATGCTGGACCACAGCAGAAGCAC-3′ and 5′-TGGCGTCTGCCGCCCGCG-3′. Primers for MPZ fragment duplication are as follows: 5′-CAATGCTGGACCACAGCAGAAGCA-3′ and 5′-CATACAGCATTGCATACATGGCGTC-3′. Cells were fixed and prepared for imaging (anti-DDK #TA50011-100 antibody from OriGene; ProLong Gold antifade reagent with DAPI #P36931) 48 h after transfection. All cell slides were imaged on a Nikon A1 inverted confocal microscope using the Galvo scan head, NIS-Elements and ImageJ software for quantification.

### Statistics and reproducibility

No statistical method was used to determine the necessary sample size for each experiment. The number of experimental replicates, representing the number of mice per genotype, is indicated in the respective legends. Data analysis was performed using GraphPad Prism version 9.5.1.733. Data are expressed as mean ± standard deviation. Comparison of the means was performed using an unpaired *t*-test for two groups and ordinary one-way or two-way ANOVA for ≥3 groups followed by Tukey–Kramer (comparison between all groups) multiple comparison test. Statistical details are described in the figure legends. Differences were declared statistically significant if *P* < 0.05. The following statistical significance indicators are used: *P < 0.05, **P < 0.005 and ^#^*P* < 0.0005.

## Results

To determine the ability of ATase inhibitors to discriminate between disease states characterized by ER proteotoxicity and those that are not, we targeted three mouse models of Charcot–Marie–Tooth disease, Pmp22^Tr−J^, C3-PMP22 and Mpz^ttrr^. Although they all develop a peripheral form of neuropathy that resembles Charcot–Marie–Tooth disease, the genetic and molecular underpinnings are very different. Pmp22^Tr−J^ mice carry a single copy of *Pmp22* with a spontaneous L16C mutation that causes mouse Pmp22 to misfold and aggregate in the ER, while C3-PMP22 mice express three copies of WT human *PMP22*, which does not aggregate in the ER.^36-42^ Consistently, when expressed in Chinese Hamster Ovary (CHO) cells, the Tr-j/trembler Jackson mutant version of human PMP22 (PMP22_Tr-J_) displayed a punctate staining that appeared to be fully sequestered in the ER while the WT version (PMP22_WT_) did not (Supplementary Fig. 1). In contrast to PMP22-based mice, Mpz^ttrr^ mice carry a spontaneous mutation of mouse Mpz. Both PMP22 and MPZ are membrane proteins that insert into the ER membrane. However, the Tr-j mutation on PMP22 resides in the lumen of the ER while the ttrr/totterer mutation on MPZ resides on the cytosolic portion of the protein (Supplementary Fig. 2). When expressed in CHO cells, the ttrr mutant version of human MPZ (MPZ_ttrr_) appeared mislocalized, as compared with the WT version (MPZ_WT_), but did not display any sequestration within the ER (Supplementary Fig. 1). This finding is consistent with the mislocalization of other C-terminal alterations of MPZ.^43^ Finally, to separate soluble and aggregated species of both mutants, MPZ_ttrr_- and PMP22_Tr-J_-expressing cells were sequentially lysed with Triton^™^ X-100 (soluble protein species) and sodium dodecyl sulfate (aggregated protein species), and the mutant proteins were analysed based on their migration profile. In contrast to MPZ_ttrr_, PMP22_Tr-J_ was almost exclusively found in the aggregated form (Supplementary Fig. 3). Therefore, Pmp22^Tr−J^, C3-PMP22 and Mpz^ttrr^ mice can be used as proof of concept to establish the selectivity of ATase1/ATase2 inhibitors towards ER-specific proteotoxicity.

**Figure 1:**
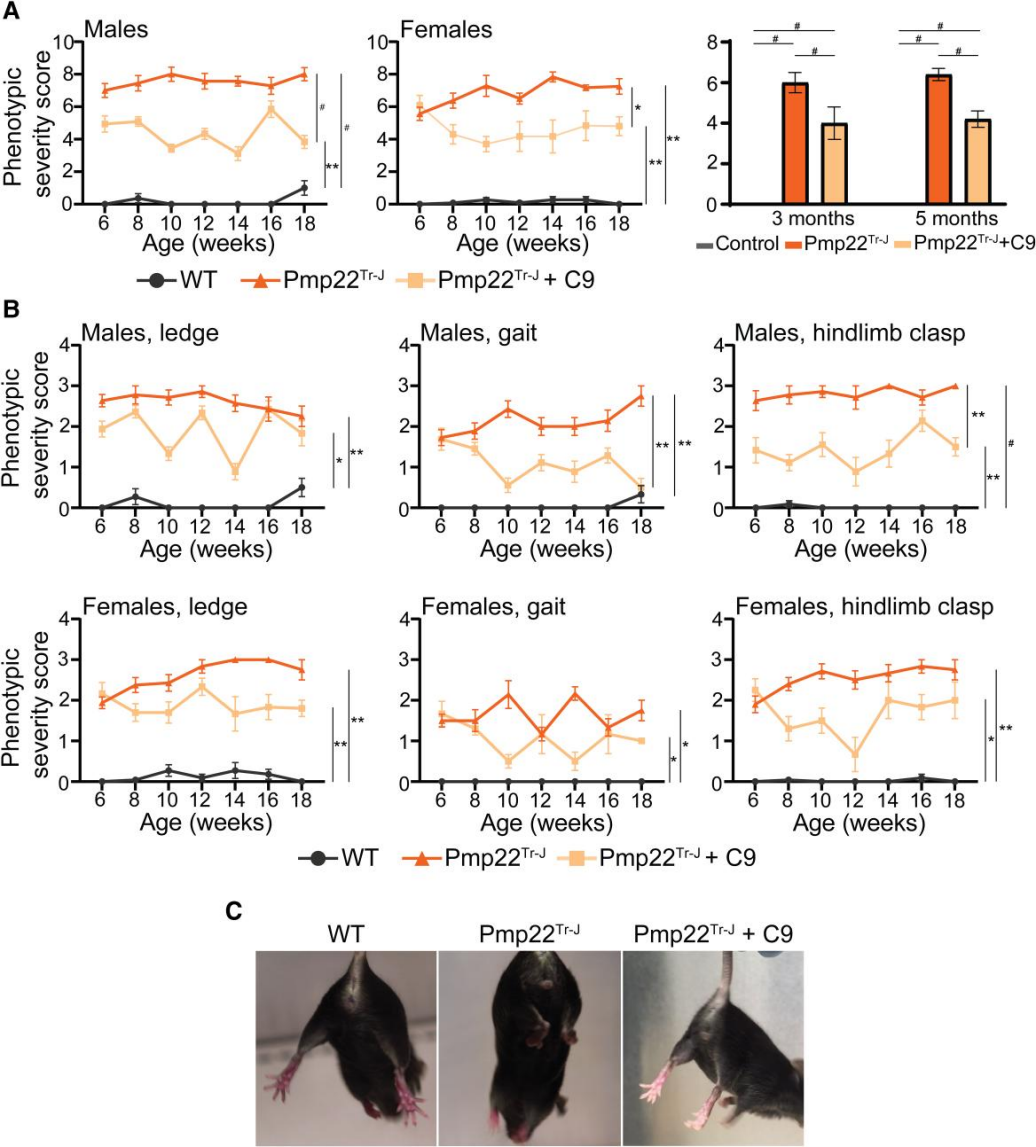
**ATase inhibition rescues the phenotypic severity score of Pmp22^Tr−J^ mice.** (**A**) Phenotypic severity score represented as a sum of ledge, gait and hindlimb clasping (*n* = 4–20 animals/group; single data points are shown in Supplementary Fig. 4). *P < 0.05, **P < 0.005 and ^#^*P* < 0.0005 via a two-way ANOVA (genotype × age; *F* males statistic = 409.8 and *F* females statistic = 270.4 for genotype factor). Histogram (*n* = 12 animals/group), ^#^*P* < 0.0005 via a two-way ANOVA (genotype × age; *F* statistic = 1256 for genotype factor). (**B**) Ledge, gait and hindlimb clasp severity score of males (upper panel) and female (lower panel; *n* = 4–24 animals/group; single data points are shown in Supplementary Fig. 4). *P < 0.05 and **P < 0.005 via a two-way ANOVA (genotype × age; *F* males statistic = 310.6:169.8:168.7 and *F* females statistic = 267.4:72.95:170.1 for ledge, gait and hindlimb clasp genotype factor, respectively). (**C**) Representative image of WT, Pmp22^Tr−J^ and Pmp22^Tr−J^ with C9 treatment animals at 5 months during the hindlimb clasp test. Figure 1 with single point graphs is shown as Supplementary Fig. 4.

**Figure 2:**
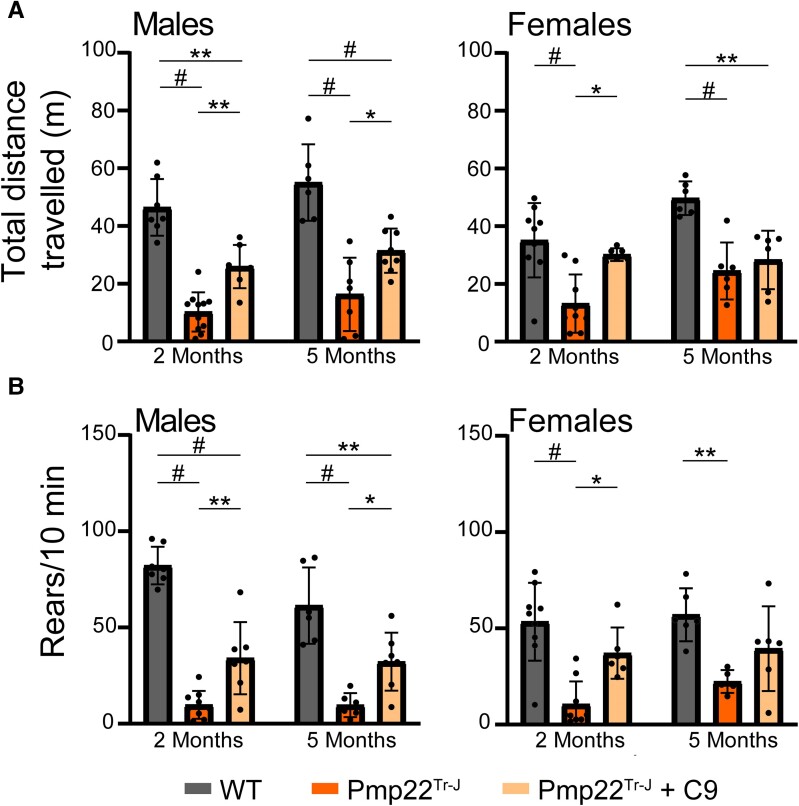
**ATase inhibition rescues spontaneous motor activity of Pmp22^Tr−J^ mice.** (**A**) Total distance travelled. Two-month-old males (WT, *n* = 7; Pmp22^Tr−J^, *n* = 11; Pmp22^Tr−J^ treated, *n* = 7), 2-month-old females (WT, *n* = 9; Pmp22^Tr−J^, *n* = 9; Pmp22^Tr−J^ treated, *n* = 6), 5-month-old males (WT, *n* = 6; Pmp22^Tr−J^, *n* = 7; Pmp22^Tr−J^ treated, *n* = 8) and 5-month-old females (*n* = 6 animals per group). *P < 0.05, **P < 0.005 and ^#^*P* < 0.0005 via a two-way ANOVA (genotype × age; *F* males statistic = 56.91 and *F* females statistic = 16.07 for genotype factor). (**B**) Rear/hind legs standing. Two-month-old males (WT, *n* = 7; Pmp22^Tr−J^, *n* = 9; Pmp22^Tr−J^ treated, *n* = 7), 2-month-old females (WT, *n* = 9; Pmp22^Tr−J^, *n* = 9; Pmp22^Tr−J^ treated, *n* = 6), 5-month-old males (WT, *n* = 6; Pmp22^Tr−J^, *n* = 6; Pmp22^Tr−J^ treated, *n* = 7) and 5-month-old females (WT, *n* = 6; Pmp22^Tr−J^, *n* = 5; Pmp22^Tr−J^ treated, *n* = 6). *P < 0.05, **P < 0.005 and ^#^*P* < 0.0005 via a two-way ANOVA (genotype × age; *F* males statistic = 72.25 and *F* females statistic = 18.79 for genotype factor).

Pmp22^Tr−J^ mice were treated with an oral formulation (50 mg/kg/day) of C9, a specific inhibitor of ATase1 and ATase2, following the well-established administration protocols.^10,12,15,20^ Treatment began at weaning (post-natal day 21), a time when the mice already displayed phenotypic deficits. The evolution of the disease phenotype was initially determined with a modified ataxia severity score (Supplementary Table 1), which examines ledge wall, hindlimb clasping and gait functions.^33,44,45^ As expected, Pmp22^Tr−J^ mice scored very poorly with a combined severity score in the 6–8 range in both sexes while WT remained stable within the 0–2 range (Fig. 1A). C9 treatment reduced the severity score in both sexes (Fig. 1A). Importantly, the improvement remained stable throughout the entire period of treatment and was manifested across all measured functions (Fig. 1A–1C; Supplementary Fig. 4). Next, we evaluated the animals for total distance travel and rear/hind paws standing frequency on the open field test battery. In both cases, C9 treatment produced a consistent and stable improvement, which was evident in both males and females and throughout the entire period of treatment (Fig. 2A and B).

**Figure 3:**
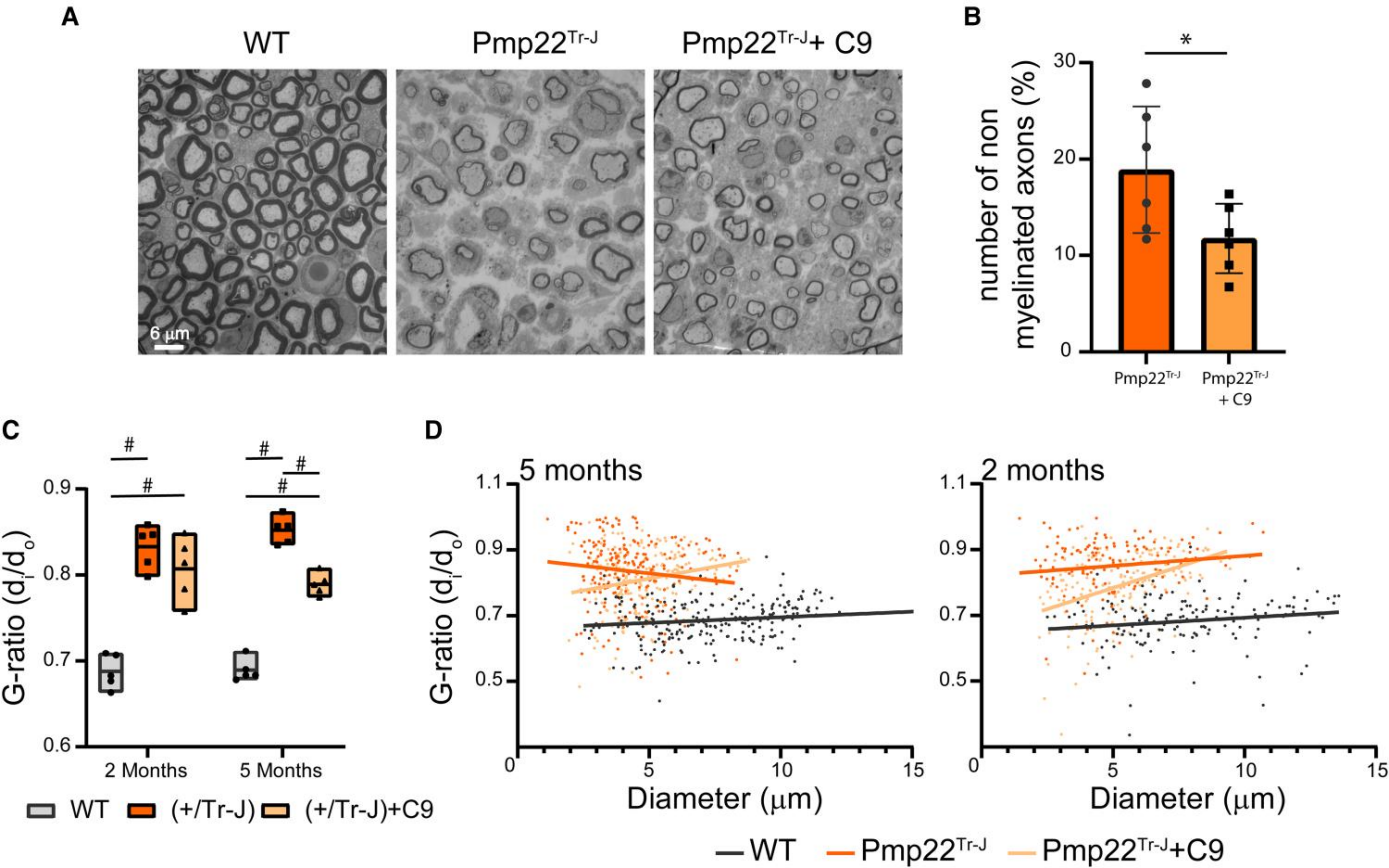
**ATase inhibition improves myelin morphology in the Pmp22^Tr−J^ mice.** (**A**) Electron micrographs of the sciatic nerves at 5 months of Pmp22^Tr−J^, Pmp22^Tr−J^ treated and WT littermates. (**B**) Percentage of non-myelinated axons (*n* = 6 animals per group; total axons per animal > 110). *P < 0.05. *t* = 2.32 via mean comparison using unpaired Student’s *t*-test. (**C**) G-ratio value representation of sciatic nerves (*n* = 5 animals/group; total axons per animal > 25). ^#^*P* < 0.0005 via a two-way ANOVA comparison (genotype × age; *F* statistic = 248.2 for genotype factor; *F* statistic = 1.19 for age factor). (**D**) G-ratio distribution according to axon diameter (*n* = 5 animals per group; total axons per animal > 25).

Post-mortem electron micrograph evaluation of the sciatic nerve revealed a marked loss of myelin in Pmp22^Tr−J^ mice (Fig. 3A). This was partially prevented by C9 treatment (Fig. 3A). Importantly, C9 treatment reduced the number of unmyelinated axons by about 40–50% (Fig. 3B) and increased the thickness of myelin sheets around myelinated axons, as manifested by the ratio of inner-to-outer axonal diameter (g-ratio; Fig. 3C and D). Again, the g-ratio rescuing effect remained stable throughout the entire period of treatment. A salient feature of C9 treatment was the restoration of myelin around smaller diameter axons (Fig. 3A; Supplementary Fig. 5).

**Figure 4:**
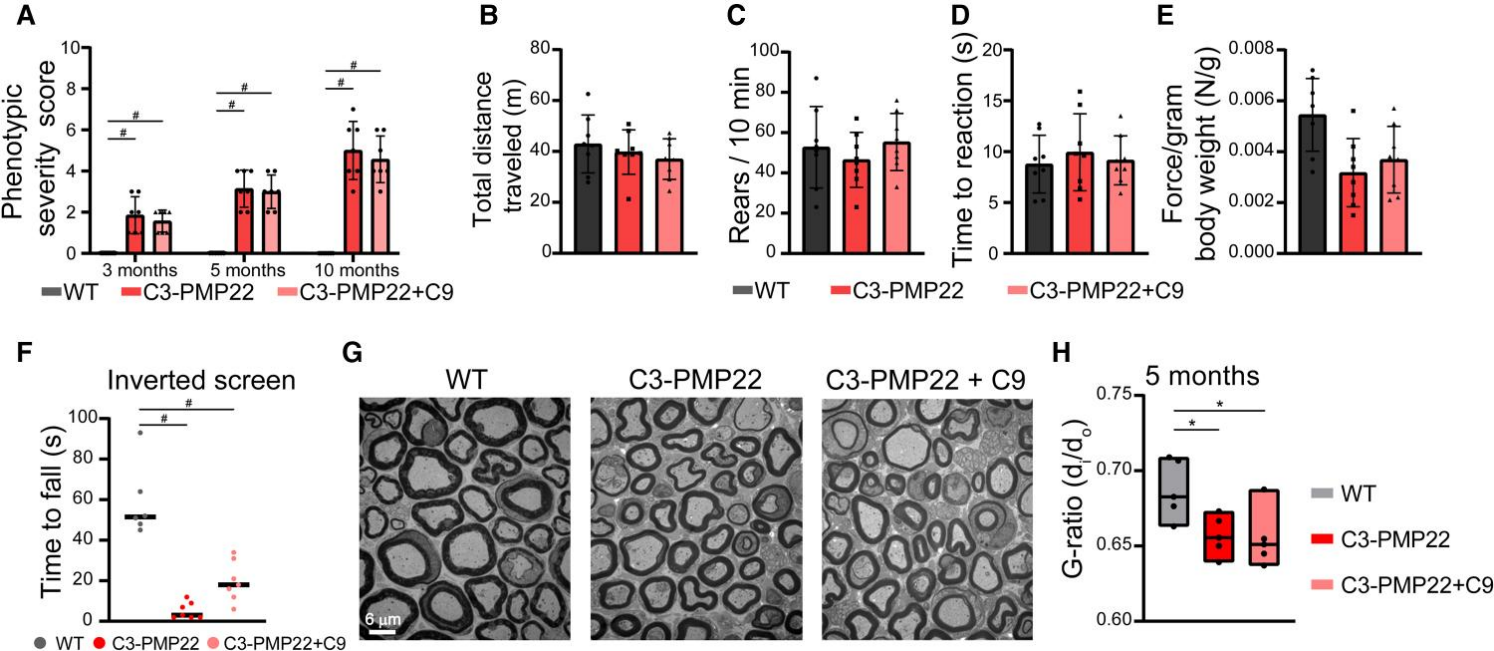
**Phenotypic assessment of C3-PMP22 mice.** (**A**) Phenotypic severity score represented as a sum of ledge, gait and hindlimb clasping at different ages (*n* = 7/group). **P < 0.005 and ^#^*P* < 0.0005 via a two-way ANOVA comparison (genotype × age; *F* statistic = 110.2 for genotype factor; *F* statistic = 33.99 for age factor). (**B**) Open field assay. Total distance travelled. Two months old (*n* = 8/group). Non-significant *P* > 0.05 via a one-way ANOVA comparison (*F* statistic = 0.7895). (**C**) Open field assay. Rears. Two months old (*n* = 8/group). Non-significant *P* > 0.05 via a one-way ANOVA comparison (*F* statistic = 0.6302). (**D**) Hot plate time to reaction. Two months old (*n* = 8/group). Non-significant *P* > 0.05 via a one-way ANOVA comparison (*F* statistic = 0.3002). (**E**) Grip strength normalized to body weight. Two months old (*n* = 8/group). Non-significant *P* > 0.05 via a one-way ANOVA comparison (*F* statistic = 3.169). (**F**) Time to fall from the inverted screen. Two months old (WT, *n* = 6; C3-PMP22, *n* = 7; C3-PMP22 treated, *n* = 7). ^#^*P* < 0.0005 via a one-way ANOVA (*F* statistic = 35.89). (**G**) Electron micrographs of the sciatic nerves at 5 months of C3-PMP22, C3-PMP22 treated and WT littermates. (**H**) G-ratio value representation of sciatic nerves (*n* = 5 animals/group; total axons per animal > 35). *P < 0.05 via a one-way ANOVA comparison (*F* statistic = 5.2).

**Figure 5:**
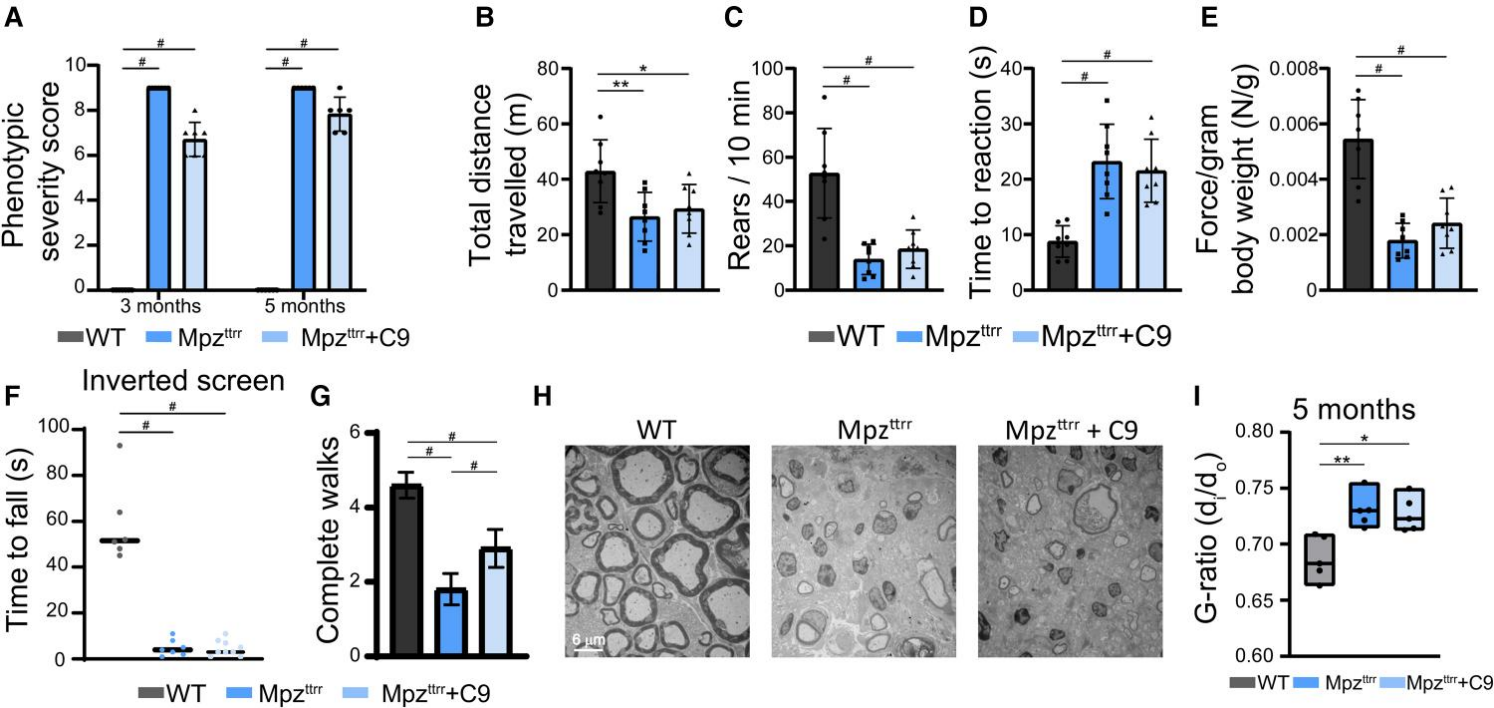
**Phenotypic assessment of Mpz^ttrr^ mice.** (**A**) Phenotypic severity score represented as a sum of ledge, gait and hindlimb clasp at different ages (WT, *n* = 7; Mpz^ttrr^, *n* = 7; Mpz^ttrr^ treated, *n* = 6). ^#^*P* < 0.0005 via a two-way ANOVA comparison (genotype × age; *F* statistic = 769.4 for genotype factor; *F* statistic = 2.2 for age factor). (**B**) Open field assay. Total distance travelled. Two months old (*n* = 8 animals/group). *P < 0.05 and **P < 0.005 via a one-way ANOVA comparison (*F* statistic = 6.56). (**C**) Open field assay. Rears. Two months old (*n* = 8 animals/group). ^#^*P* < 0.0005 via a one-way ANOVA comparison (*F* statistic = 20.51). (**D**) Hot plate time to reaction. Two months old (*n* = 8 animals/group). ^#^*P* < 0.0005 via mean one-way ANOVA comparison (*F* statistic = 17.45). (**E**) Grip strength normalized to body weight. Two months old (*n* = 8 animals/group). ^#^*P* < 0.0005 via a one-way ANOVA comparison (*F* statistic = 28.41). (**F**) Time to fall from the inverted screen. Two months old (WT, *n* = 6; Mpz^ttrr^, *n* = 7; Mpz^ttrr^ treated, *n* = 9). ^#^*P* < 0.0005 via a one-way ANOVA (*F* statistic = 68.21). (**G**) Number of complete walks through the balance beam (*n* = 10 animals/group). ^#^*P* < 0.0005 via a one-way ANOVA (*F* statistic = 106.8). (**H**) Electron micrographs of the sciatic nerves at 5 months of Mpz^ttrr^, Mpz^ttrr^ treated and WT littermates. (**I**) G-ratio value representation of sciatic nerves (*n* = 5 animals/group; total axons per animal > 35. *P < 0.05 and **P < 0.005 via a one-way ANOVA comparison (*F* statistic = 9.82).

In contrast to Pmp22^Tr−J^ mice, ATase inhibition of C3-PMP22 or Mpz^ttrr^ mice did not elicit phenotypic improvement (Figs. 4 and 5). The Charcot–Marie–Tooth disease–like phenotype of Mpz^ttrr^ mice developed earlier than C3-PMP22 mice and was much more severe, with marked defects across different motor-based paradigms. The different phenotypic severity was manifested at the histological level with Mpz^ttrr^ mice displaying a drastic loss of neurons and myelin (Figs. 4 and 5). Importantly, no rescuing effect was observed following treatment with C9 (Figs. 4 and 5).

In conclusion, the inhibition of the ATases by C9 partially rescued both the behavioural and pathological features associated with the Charcot–Marie–Tooth disease–like phenotype of Pmp22^Tr−J^ mice but was not able to modify the progression of the disease in C3-PMP22 or Mpz^ttrr^ mice.

## Discussion

ATase1 and ATase2 are type II ER-resident membrane proteins with the catalytic domain facing the lumen of the organelle.^18^ They use acetyl-CoA, imported into the ER lumen by AT-1/SLC33A1, to acetylate ER cargo and resident proteins.^17^ Genetic disruption of either *Atase1* or *Atase2* in the mouse stimulates reticulophagy.^14^ Biochemical inhibition of the Atases in the mouse also stimulates reticulophagy.^10,15,20^ Finally, reduced import of acetyl-CoA into the ER lumen stimulates reticulophagy by limiting the catalytic activity of the ATases.^9,10,17,46^ Both cell- and animal-based studies indicate that the regulation of reticulophagy downstream of the ATases depends on the acetylation status of ATG9A. Specifically, acetylated ATG9A prevents reticulophagy while non-acetylated ATG9A stimulates reticulophagy.^10,12-14,19^ Importantly, the acetylation of ATG9A occurs in the lumen of the ER.^10,12-14,19^ In essence, the ER acetylation machinery appears to be spatially positioned to act as a novel branch of the ER quality control system to help disposing of protein aggregates that form within the ER lumen.

Inhibition of the ATases was able to rescue the progeria-like phenotype of AT-1 sTg and SLC13A5 sTg, two mouse models of ER hyperacetylation.^12,15,20^ Inhibition of the ATases was also able to resolve the Alzheimer’s disease–like phenotype of APP_695/swe_ and APP_695/swe_/PS1-dE9 mice.^10,20^ Importantly, ATase inhibition did not resolve the disease phenotype of mHtt^Q160^ mice, a model of Huntington disease, or hSOD1^G93A^ mice, a model of amyotrophic lateral sclerosis.^10^ APP is a type I membrane protein that inserts into the ER to engage the secretory pathway, while Htt and SOD1 are cytosolic proteins and do not engage the secretory pathway. Furthermore, the proteotoxic aggregates of the A53T mutant version of α-synuclein, which is associated with an autosomal dominant form of Parkinson’s disease, were successfully degraded by reduced ER acetylation only when α-synuclein was forced to insert into the ER lumen by adding a signal peptide to its N-terminus.^10^ In essence, ATase inhibitors appear to be selective for ER/secretory pathway proteotoxicity.

This study adds an additional layer of evidence by showing that misfolding states that occur in the lumen of the ER, and that are associated with aberrant aggregation of the misfolded polypeptide in the ER, can be targeted by ATase inhibitors, while misfolding states that occur elsewhere, or that do not yield protein aggregates within the ER, cannot. In essence, work conducted with different mouse models of human diseases has established the ATases as the first proven targets for proteotoxic states that initiate within the lumen of the ER (see present study and Peng *et al*., Rigby *et al*., Fernandez-Fuente *et al*. and Murie *et al*.^10,12,14,15,20^). This selectivity provides encouraging hopes for many hereditary diseases caused by mutations that force the protein to misfold and aggregate within the ER and secretory pathway, such as Charcot–Marie–Tooth disease, Pelizaeus–Merzbacher disease and cystic fibrosis. It also provides a way to limit or completely avoid unwanted effects caused by non-selective global activation of autophagy. This selectivity, however, also requires a personalized form of medicine with careful selection of patients, particularly for those diseases, such as hereditary forms of neuropathy, where different genetic events (i.e. mutation, duplication or deletion) can be the underlying pathogenic mechanism. Using Charcot–Marie–Tooth disease as a test case, our data would indicate that ATase inhibition may not be effective in patients with *PMP22* duplication (classified as CMT1A) but may be effective in patients with *PMP22* mutations (classified as CMT1E). Although treatment of mice with the *Mpz_ttrr_* mutation was ineffective, there are a variety of dominant *MPZ* mutations (classified as CMT1B) that are associated with the misfolding of the ER luminal portion of the protein, ER retention and even ER aggregation together with activation of the unfolded protein response.^47,48^ These forms of Charcot–Marie–Tooth disease may be responsive to ATase inhibition and stimulation of reticulophagy.

## Supplementary Material

fcae232_Supplementary_Data

## Data Availability

Source data for the graphs and charts are available as Supplementary material, and any remaining information can be obtained from the corresponding author upon reasonable request.
