# Supplementary material for: Spatial selectivity of ATase inhibition in mouse models of Charcot–Marie–Tooth disease
Source: Brain Commun. 2024 Jul 9;6(4):fcae232. doi: 10.1093/braincomms/fcae232 (PMC11258571; doi:10.1093/braincomms/fcae232)
Supplement: fcae232_Supplementary_Data [file fcae232_supplementary_data.zip › Original Submission.pdf]

OXFORD UNIVERSITY PRESS | Brain Communications

Spatial selectivity of ATase inhibition in mouse models of Charcot-Marie-Tooth disease

Journal:	*Brain Communications*
Manuscript ID	BRAINCOM-2024-121
Manuscript Type:	Original Article
Date Submitted by the Author:	22-Feb-2024

Complete List of Authors:	Fernandez-Fuente, Gonzalo; University of Wisconsin-Madison, Department of Medicine; University of Wisconsin-Madison<br>Farrugia, Mark A.; University of Wisconsin-Madison, Department of Medicine; University of Wisconsin-Madison<br>Peng, Yajing; University of Wisconsin-Madison, Department of Medicine; University of Wisconsin-Madison<br>Schneider, Andrew; University of Wisconsin-Madison<br>Svaren, John; University of Wisconsin-Madison<br>Puglielli, Luigi; University of Wisconsin-Madison, Medicine

Keywords:	ATase, acetylation, proteostasis, endoplasmic reticulum, Charcot-Marie-Tooth disease

SCHOLARONE™
Manuscripts

Spatial selectivity of ATase inhibition in mouse models of Charcot-Marie-Tooth disease

Gonzalo Fernandez-Fuente[1,2,†], Mark A. Farrugia[1,2,†,#], Yajing Peng[1,2,$], Andrew Schneider[2], John Svaren[2,3], Luigi Puglielli[1,2,4*]

[†]**GF-F and MAF contributed equally to this paper**

For Review Only

Abstract

The endoplasmic reticulum (ER) acetylation machinery has emerged as a new branch of the larger ER quality control system. It regulates the selection of correctly folded polypeptides as well as reticulophagy-mediated removal of toxic protein aggregates with the former being a particularly important aspect of the proteostatic functions of ER acetylation. Essential to this function is the Nε-lysine acetyltransferase activity of ATase1 and ATase2, which regulate the induction of ER-specific autophagy though the acetylation of the autophagy protein ATG9A. Here, we used three mouse models of Charcot-Marie-Tooth disease, $Pmp22^{Tr\text{-}J}$, C3-PMP22, and Mpz^{ttrr}, to study spatial and translational selectivity of ATase inhibitors. The results show that ATase inhibition selectively targets misfolding/pro-aggregating events occurring in the lumen of the ER. Therefore, they establish the ATases as the first proven targets for disease-causing proteotoxic states that initiate within the lumen of the ER/secretory pathway.

Authors affiliations:

1 Department of Medicine, School of Medicine and Public Health, University of Wisconsin-Madison, Madison, WI 53705, USA

2 Waisman Center, University of Wisconsin-Madison, Madison, WI 53705, USA

3 Department of Comparative Biosciences, School of Veterinary Medicine, University of Wisconsin-Madison, Madison, WI 53706, USA

4 Geriatric Research Education Clinical Center, Veterans Affairs Medical Center, Madison, WI 53705, USA

# Current affiliation: Department of Biochemistry & Molecular Biology, College of Natural Science, Michigan State University, East Lansing, MI, 48824

$ Current affiliation: Wisconsin State Laboratory of Hygiene, University of Wisconsin-Madison, Madison, WI 53705, USA

*Correspondence: Luigi Puglielli, University of Wisconsin-Madison, Waisman Center, 1500 Highland Ave, Madison, WI 53705, USA

lp1@medicine.wisc.edu

For Review Only

Running title: ATase inhibition in CMT disease

Keywords: ATase; acetylation; proteostasis; endoplasmic reticulum; Charcot-Marie-Tooth disease

Abbreviations: AT-1: acetyl-CoA transporter 1; ATase1: acetyltransferase 1; ATase2 : acetyltransferase 2; ATG9A : autophagy protein 9A; C9 : compound 9; CMT : Charcot-Marie-Tooth; ER = endoplasmic reticulum; HMN : hereditary motor neuropathy; HSAN : hereditary sensory and autonomic neuropathy; MPZ : myelin protein zero; PMP22 : peripheral myelin protein 22; SPG : spastic paraplegia

Introduction

A fundamental task of the endoplasmic reticulum (ER) is to make proteins that can then engage the secretory pathway to reach their final destination within the cell or be secreted to the extracellular *milieu*. Quality control mechanisms are in place to ensure that only correctly folded polypeptides can leave the ER. Quality control mechanisms are also in place to remove misfolded/unfolded polypeptides that would otherwise accumulate in the ER and cause proteotoxicity. [1,2] By ensuring the continuous and efficient removal of toxic protein aggregates, the ER-specific autophagy (alternatively referred to as reticulophagy, ER-phagy or ER associated

degradation type II) represents a fundamental component of the ER quality control system. Defective removal of toxic protein aggregates through autophagy has been linked to different diseases across lifespan. [3-6] As such, it is not surprising that improving normal proteostatic mechanisms represents an active target for biomedical research.

The ER acetylation machinery has emerged as a novel branch of the larger ER quality control system. It regulates the selection of correctly folded polypeptides, but also reticulophagy-mediated removal of toxic protein aggregates. [7-16] ER acetylation is ensured by AT-1/SLC33A1, a membrane transporter that allows entry of cytosolic acetyl-CoA into the ER lumen, and ATase1/NAT8B and ATase2/NAT8, two ER-based acetyl-CoA:lysine acetyltransferases that use acetyl-CoA to acetylate ER cargo proteins within the ER lumen following their initial tertiary-state folding. [7,16-18]

The removal of toxic protein aggregates through reticulophagy is a particularly important aspect of the proteostatic functions of ER acetylation. This process requires ATase1- and ATase2-mediated acetylation of the ER-bound autophagy protein ATG9A, which in turn regulates the recruitment of the autophagy core machinery. [13,19] In the mouse, reduced ER acetylation causes increased induction of reticulophagy, while increased ER acetylation has the opposite effect. [9,12,20] Genetic disruption or biochemical inhibition of the ATases results in activation of reticulophagy, as well as rescue of disease-associated proteotoxicity. [10,12,14,15,21] Disease causing mutations in genes involved in the regulation of the proteostatic functions of the ER acetylation machinery (i.e.; *AT-1/SLC33A1*, *FAM134B* and *HSPB1*) have been associated with different forms of hereditary sensory and autonomic neuropathy/hereditary motor neuropathy (HSAN/HMN) and spastic paraplegias (SPGs). [22-27] Finally, HSANs/HMNs-causing mutations have been associated with mislocalization of ATG9A and impaired autophagic degradation of pathogenic aggregates. [28,29]

In terms of translational output for autophagy-based strategies, a fundamental need is to selectively target autophagy to a specific cellular location. Studies conducted in different mouse models of proteotoxicity indicate that increasing reticulophagy through targeted inhibition of the ATases is a valid strategy to rescue disease-causing proteotoxic states of the ER and secretory pathway. [10,12,14,15,21] However, these studies were not designed to discriminate between disease-causing events that force a target protein to misfold/aggregate (i.e.; a mutation) and disease-causing events that do not (i.e.; a gene duplication); or between misfolding events that affect the luminal *vs* the cytosolic portion of ER-bound membrane proteins. This is a particularly important

For Review Only

aspect of "personalized medicine" since different genetic events (i.e., mutation, duplication, or deletion) can be the underlying pathogenic mechanism. Sometimes, these different genetic events are associated with the same group of diseases (i.e.; HSAN/HMN/SPGs), or even target the same protein (i.e.; peripheral myelin protein 22, PMP22). [30,31]

Here, we used three mouse models of Charcot-Marie-Tooth (CMT) disease, a peripheral form of neuropathy, to target the above limitations. The effectiveness of targeting ATase activity was tested using neuropathy models that involve duplication or misfolding mutations of two abundant peripheral myelin proteins in Schwann cells of peripheral nerves. The results show that ATase inhibition selectively targets misfolding/pro-aggregating events occurring in the lumen of the ER, thus establishing the ATases as the first proven targets for proteotoxic states that initiate within the lumen of the ER.

For Review Only

Materials and methods

Animals

All the animals used in this study are *Mus musculus* obtained from the Jackson Laboratory. Strains B6.Cg-Tg(PMP22)C3Fbas/J (#JAX:030052), B6.D2-$Pmp22^{Tr-J}$/J (#JAX:002504) and B6.Cg-Mpz^{ttrr}/GrsrJ (#JAX:010494) were maintained on the C57BL/6J (#JAX:000664) genetic background. B6.Cg-Tg(PMP22)C3Fbas/J and B6.D2-$Pmp22^{Tr-J}$/J mice were bred as heterozygous while B6.Cg-Mpz^{ttrr}/GrsrJ were bred as homozygous.

Mice were housed in standard cages provided by the University Laboratory Animal Resources and grouped with littermates, 1-5 per cage; animals were supplied with standard chow and water *ad libitum*. The diet with Compound 9 (1 mg/g) was manufactured by Bio-Serv. [12,15,21] Treatment with Compound 9 began at weaning and continued throughout the entire life of the animals. All animal experiments were performed in accordance with the National Institute of Health Guide for the Care and Use of Laboratory Animals and were approved by the Institutional Animal Care and Use Committee of the University of Wisconsin-Madison (protocol #M005120). Wild type littermates were used as controls throughout the study. The specific age and sex of animals at time of experimentation are specified in the figure and figure legends. Genotyping from tail DNA was performed at weaning by TransnetYX using real time PCR.

Behavior testing

All behavioral assays were conducted within the Waisman Center Behavioral Testing Service (Madison, WI, USA). The experimenter was blind to the genotype of the mice during testing. All mice received a minimum of 30 minutes acclimation time to the testing room prior to each behavior assay.

Phenotypic severity score

We used a simple composite phenotype scoring system for evaluating mouse models of cerebellar ataxia described previously. [32] Blind analysis of animals was performed for three different tests: ledge, hindlimb clasping, and gait. Individual measures are scored on a scale of 0 to 3, with 0 representing an absence of the relevant phenotype and 3 representing the most severe manifestation (**Supplementary Table 1**).

Open field exploration

Open field exploration sessions lasted 30 minutes and each mouse received 1 session. Each mouse was removed from its home cage and placed in the center of the arena. The Omnitech Fusion system used photobeams to continuously monitor and record the animal's placement during the session. Testing variables included total distance travelled (in centimeters) and vertical activity episode count (rears). Data was recorded using the Omnitech Fusion system.

Hot plate

Mice were placed on a hot plate (Columbus Instruments, Hot plate analgesia meter) to evaluate the reaction time. The reaction time was scored when the animal jumped or licked its paws. A cutoff of 40 seconds was used to avoid any paw damage. Five reaction times were determined for each mouse with a latency of at least 15 min apart between measurements. Results shown for each animal are the average of the middle three values.

Grip strength

Grip strength was measured using an Ametek Chatillon DFE II (Columbus instruments, Grip strength meter). The mouse was held by the base of the tail above a wire bar connected to the force gauge. The mouse was dangled in a position that allowed it to reflexively grasp the wire with its forepaws. The investigator then pulled the animal up from the bar at a constant speed and the maximum force generated just before the mouse lost its grasp was recorded. This was repeated

For Review Only

five times for each animal. Results shown for each animal are the average of the middle three values.

Inverted screen

Mice were removed from the home cage and placed on top of the screen. A gently shake of the screen was performed to make sure the mouse had gripped the screen. Then, the screen was carefully inverted at 30 cm over the empty cage so that the mouse was upside down on the screen. Time was measured from the inversion moment to record the time latency to fall. This was repeated five times for each animal with a latency of at least 10 min between measurements. Results shown for each animal are the average of the middle three values.

Balance beam

The balance beam apparatus is composed of one smooth, plastic beam 70 cm in length and 3 cm in diameter. The beam is securely suspended 25 cm above the surface. Enclosed safe house is placed at the escape end of the beam and bedding is added to encourage the mouse to enter. A training session was conducted the day before the test session. The test session consists of five runs per animal and results are shown as total successful runs per animal (0 to 5 score).

Electron microscopy

Following CO_2 euthanasia, sciatic nerve was extracted and fixed in 2.5% glutaraldehyde in 0.1 M phosphate buffer (PB) overnight at 4 °C. Fixed samples were rinsed 5 x 5 min in PB and post-fixed in 1% osmium tetroxide, 1% potassium ferrocyanide in 0.1 M PB for 1 hour at room temperature, then rinsed in PB. Dehydration was performed in ethanol series (35%, 50%, 70%, 80%, 90% for 10 min each step, 95% for 20 min, 100% for 2 x 10 min) at room temperature and 100% ethanol at 4 °C overnight, then transitioned in propylene oxide (PO) 2 x 7 min at room temperature. Fully dehydrated samples were infiltrated in increasing concentrations of PolyBed 812 (Polysciences Inc.) and PO mixtures. Embedding and polymerization occurred in fresh PolyBed 812 for 24 h at 60 °C. The samples were sectioned on a Leica EM UC6 ultra-microtome at 100 nm, collected on formvar-coated 2 x 1 mm slot Cu grids (EMS Hatfield, PA) and post-stained with uranyl acetate and lead citrate. The sectioned samples were viewed at 80 kV on a Philips CM120 transmission electron microscope equipped with AMT BioSprint12 digital camera (AMT Imaging Systems).

For Review Only

Morphometry

Non-overlapping electron micrographs of sciatic nerves were analyzed for axon diameter and g-ratio. A minimum of 100 randomly selected fibers were analyzed per animal using the g-ratio plug-in of the ImageJ software, which allowed for semiautomated analysis of randomly selected sets of fibers. [33] For each nerve, the percentage of non-myelinated axons was calculated by direct count from the EM micrographs.

Cell culture and plasmids

CHO-K1 (Ovary Chinese Hamster, ATCC CCL-61™) cells were grown in Dulbecco's modified Eagle's medium (DMEM; Corning #10-017-CV) supplemented with 10% Fetal Bovine Serum (Corning #35-010-CV) and 1% penicillin/streptomycin/glutamine (Gibco #10378016). Cells were maintained at 37 °C in a humidified atmosphere with 5% CO_2. Cells were transiently transfected with Lipofectamine 2000 (Invitrogen #11668019) using the following constructs: mCherry-ER-3 plasmid, a gift from Michael Davidson (Addgene plasmid # 55041); PMP22 Human Tagged ORF Clone (Origene #RC216500); MPZ Human Tagged ORF Clone (Origene #RC202450). Additionally, plasmids for human $PMP22_{Tr-J}$ (T47C, Leu to Pro) and human MPZ_{ttrr} (GTGC deletion and TGTATGCAATGC duplication) were developed in our laboratory using site-directed mutagenesis (New England BioLabs Q5 Site-Directed Mutagenesis Kit #E0554S). Primers for Tr-J mutation; 5'-GTCGCGGTGCCGGTGCTGCTG-3', and 5'-GTGGAGGACGATGATACTCAGCAAC-3'. Primers for MPZ fragment deletion; 5'-TGTATGCAATGCTGGACCACAGCAGAAGCAC-3', and 5'-TGGCGTCTGCCGCCCGCG-3'. Primers for MPZ fragment duplication; 5'-CAATGCTGGACCACAGCAGAAGCA-3', and 5'-CATACAGCATTGCATACATGGCGTC-3'. Cells were fixed and prepared for imaging (Anti-DDK #TA50011-100 antibody from Origene; ProLong Gold antifade reagent with DAPI #P36931) 48 hours after transfection. All cell slides were imaged on a Nikon A1 inverted confocal microscope using NIS-Elements software and the Galvano scan head.

Statistics and reproducibility

For Review Only

No statistical method was used to determine the necessary sample size for each experiment. The number of experimental replicates, representing the number of mice per genotype, is indicated in the respective legends. A minimum of $n = 5$ animals were used throughout our study, as required by the experimental design. Data analysis was performed using GraphPad Prism version 9.5.1.733. Data are expressed as mean ± standard deviation (SD) unless otherwise specified. Comparison of the means was performed using an unpaired *t*-test for two groups and ordinary one-way or two-way ANOVA for ≥ 3 groups followed by Tukey-Kramer (comparison between all groups) multiple comparisons test. Statistical test details are described in the figure legends. Differences were declared statistically significant if $P < 0.05$ and the following statistical significance indicators are used: * $P < 0.05$; ** $P < 0.005$; $^{\#}$ $P < 0.0005$.

For Review Only

Data availability

All data supporting the findings of this study are available within the paper and its Supplementary Data/Tables.

Results

To determine the ability of ATase inhibitors to discriminate between disease states characterized by ER proteotoxicity and those that are not, we targeted three mouse models of CMT disease, Pmp22^{Tr-J}, C3-PMP22, and Mpz^{ttrr}. Although they all develop a peripheral form of neuropathy that resembles CMT disease, the genetic and molecular underpinnings are very different. Pmp22^{Tr-J} mice carry a single copy of *Pmp22* with a spontaneous L16C mutation that causes mouse Pmp22 to misfold and aggregate in the ER, while C3-PMP22 mice express three copies of wild-type human *PMP22*, which does not aggregate in the ER. [34-40] Consistently, when expressed in Chinese Hamster Ovary (CHO) cells, the Tr-j/trembler Jackson mutant version of human PMP22 (PMP22$_{Tr-J}$) displayed a punctate staining that appeared to be fully sequestered in the ER while the WT version (PMP22$_{WT}$) did not (**Supplementary Figure 1A**). In contrast to PMP22-based mice, Mpz^{ttrr} mice carry a spontaneous mutation of mouse Mpz. Both PMP22 and MPZ are membrane proteins that insert into the ER membrane. However, the Tr-j mutation on PMP22 resides in the lumen of the ER while the ttrr/totterer mutation on MPZ resides on the

cytosolic portion of the protein (**Supplementary Figure 2**). When expressed in CHO cells, the ttrr mutant version of human MPZ (MPZ_{ttrr}) appeared mislocalized, as compared to WT version (MPZ_{WT}), but did not display any sequestration within the ER (**Supplementary Figure 1B**). This finding is consistent with mislocalization of other C-terminal alterations of MPZ. [41] Therefore, $Pmp22^{Tr-J}$, C3-PMP22, and Mpz^{ttrr} mice can be used as proof-of-concept to establish the selectivity of ATase1/ATase2 inhibitors towards ER-specific proteotoxicity.

$Pmp22^{Tr-J}$ mice were treated with an oral formulation (50 mg/kg/day) of compound 9 (C9), a specific inhibitor of ATase1 and ATase2, following well established administration protocols. [10,12,15,21] Treatment began at weaning (post-natal day 21), a time when the mice already displayed phenotypic deficits. The evolution of the disease phenotype was initially determined with a modified ataxia severity score (**Supplementary Table 1**), which examines ledge wall, hindlimb clasping, and gait functions. [32,42,43] As expected, $Pmp22^{Tr-J}$ mice scored very poorly with a combined severity score in the 6-8 range in both sexes while WT remained stable within the 0-2 range (**Figure 1A**). C9 treatment reduced the severity score in both sexes (**Figure 1A**). Importantly, the improvement remained stable throughout the entire period of treatment and was manifested across all measured functions (**Figure 1A-1C**). Next, we evaluated the animals for total distance travel and rear/hind paws standing frequency on the open field test battery. In both cases C9 treatment produced a consistent and stable improvement, which was evident in both males and females, and throughout the entire period of treatment (**Figure 2A-2B**).

Post-mortem electron micrograph evaluation of the sciatic nerve revealed a marked loss of myelin in $Pmp22^{Tr-J}$ mice (**Figure 3A**). This was partially prevented by C9 treatment (**Figure 3A**). Importantly, C9 treatment reduced the number of unmyelinated axons by about 40 to 50% (**Figure 3B**) and increased the thickness of myelin sheets around myelinated axons, as manifested by the ratio of inner-to-outer axonal diameter (g-ratio; **Figure 3C-3D**). Again, the g-ratio rescuing effect remained stable throughout the entire period of treatment. A salient feature of C9 treatment was the restoration of myelin around smaller diameter axons (**Figure 3A and Supplementary Figure 3**).

In contrast to $Pmp22^{Tr-J}$ mice, ATase inhibition of C3-PMP22 or Mpz^{ttrr} mice did not elicit phenotypic improvement (**Figure 4** and **Figure 5**). The CMT-like phenotype of Mpz^{ttrr} mice developed earlier than C3-PMP22 mice and was much more severe, with marked defects across different motor-based paradigms. The different phenotypic severity was manifest at the

For Review Only

histological level with Mpz^{ttrr} mice displaying a drastic loss of neurons and myelin (**Figure 4** and **Figure 5**). Importantly, no rescuing effect was observed following treatment with C9 (**Figure 4** and **Figure 5**).

In conclusion, the inhibition of the ATases by C9 partially rescued both the behavioral and pathological features associated with the CMT-like phenotype of $Pmp22^{Tr\text{-}J}$ mice but was not able to modify the progression of the disease in C3-PMP22 or Mpz^{ttrr} mice.

Discussion

ATase1 and ATase2 are type II ER-resident membrane proteins with the catalytic domain facing the lumen of the organelle. [18] They use acetyl-CoA, imported into the ER lumen by AT-1/SLC33A1, to acetylate ER cargo and resident proteins. [17] Genetic disruption of either *Atase1* or *Atase2* in the mouse stimulates reticulophagy. [14] Biochemical inhibition of the Atases in the mouse also stimulates reticulophagy. [10,15,21] Finally, reduced import of acetyl-CoA into the ER lumen stimulates reticulophagy by limiting the catalytic activity of the ATases. [9,10,17,44] Both cell- and animal-based studies indicate that the regulation of reticulophagy downstream of the ATases depends on the acetylation status of ATG9A. Specifically, acetylated ATG9A prevents reticulophagy while non-acetylated ATG9A stimulates reticulophagy. [10,12-14,19] Importantly, the acetylation of ATG9A occurs in the lumen of the ER. [10,12-14,19] In essence, the ER acetylation machinery appears to be spatially positioned to act as a novel branch of the ER quality control system to help disposing of protein aggregates that form within the ER lumen.

Inhibition of the ATases was able to rescue the progeria-like phenotype of AT-1 sTg and SLC13A5 sTg, two mouse models of ER hyperacetylation. [12,15,21] Inhibition of the ATases was also able to resolve the Alzheimer's disease-like phenotype of $APP_{695/swe}$ and $APP_{695/swe}$/PS1-dE9 mice. [10,21] Importantly, ATase inhibition did not resolve the disease phenotype of $mHtt^{Q160}$ mice, a model of Huntington disease, or $hSOD1^{G93A}$ mice, a model of amyotrophic lateral sclerosis. [10] APP is a type I membrane protein that inserts into the ER to engage the secretory pathway, while Htt and SOD1 are cytosolic proteins and do not engage the secretory pathway. Furthermore, the proteotoxic aggregates of the A53T mutant version of α-synuclein, which is associated with an autosomal dominant form of Parkinson's disease, were successfully degraded by reduced ER acetylation only when α-synuclein was forced to insert into the ER lumen by adding a signal

For Review Only

peptide to its N-terminus. [10] In essence, ATase inhibitors appear to be selective for ER/secretory pathway proteotoxicity.

This study adds an additional layer of evidence by showing that misfolding states that occur in the lumen of the ER, and that are associated with aberrant aggregation of the misfolded polypeptide in the ER, can be targeted by ATase inhibitors, while misfolding states that occur elsewhere, or that do not yield protein aggregates within the ER, cannot. In essence, work conducted with different mouse models of human diseases has established the ATases as the first proven targets for proteotoxic states that initiate within the lumen of the ER (see present study and [10,12,14,15,21]). This selectivity provides encouraging hopes for many hereditary diseases caused by mutations that force the protein to misfold and aggregate within the ER and secretory pathway, such as CMT, Pelizaeus-Merzbacher disease and cystic fibrosis, among others. It also provides a way to limit or completely avoid unwanted effects caused by non-selective global activation of autophagy. This selectivity, however, also requires a personalized form of medicine with careful selection of patients, particularly for those diseases, such as hereditary forms of neuropathy, where different genetic events (i.e., mutation, duplication, or deletion) can be the underlying pathogenic mechanism. Using CMT as a test case, our data would indicate that ATase inhibition may not be effective in patients with *PMP22* duplication (classified as CMT1A) but may be effective in patients with *PMP22* mutations (classified as CMT1E). Although treatment of mice with the Mpz_{ttrr} mutation was ineffective, there are a variety of dominant *MPZ* mutations (classified as CMT1B) that are associated with misfolding of the ER luminal portion of the protein, ER retention and even ER aggregation together with activation of the unfolded protein response. [45,46] These forms of CMT may be responsive to ATase inhibition and stimulation of reticulophagy.

For Review Only

Funding

The Puglielli laboratory is supported by the NINDS (R01NS094154), the NIGMS (R01GM148487), the NIA (R01AG078794) and the Charcot-Marie-Tooth Association. The Svaren laboratory is supported by the NINDS (R01NS130566) and the Charcot-Marie-Tooth Association. This research also benefitted from a core grant to the Waisman Center from NICHD-U54 HD090256.

Competing interests

The authors report no competing interests to disclose.

Supplementary material

Supplementary material is available at *Brain Communications* online.

References

1. Buchberger A, Bukau B, Sommer T. Protein quality control in the cytosol and the endoplasmic reticulum: brothers in arms. *Mol Cell.* 2010;40(2):238-252.
2. Trombetta ES, Parodi AJ. Quality control and protein folding in the secretory pathway. *Annu Rev Cell Dev Biol.* 2003;19:649-676.
3. Frake RA, Ricketts T, Menzies FM, Rubinsztein DC. Autophagy and neurodegeneration. *J Clin Invest.* 2015;125(1):65-74.
4. Levine B, Packer M, Codogno P. Development of autophagy inducers in clinical medicine. *J Clin Invest.* 2015;125(1):14-24.
5. Nixon RA. The role of autophagy in neurodegenerative disease. *Nature medicine.* 2013;19(8):983-997.
6. Mizushima N, Levine B, Cuervo AM, Klionsky DJ. Autophagy fights disease through cellular self-digestion. *Nature.* 2008;451(7182):1069-1075.
7. Farrugia MA, Puglielli L. Nepsilon-lysine acetylation in the endoplasmic reticulum - a novel cellular mechanism that regulates proteostasis and autophagy. *J Cell Sci.* 2018;131(22).
8. Dieterich IA, Cui Y, Braun MM, et al. Acetyl-CoA flux from the cytosol to the ER regulates engagement and quality of the secretory pathway. *Sci Rep.* 2021;11(1):2013.
9. Peng Y, Li M, Clarkson BD, et al. Deficient Import of Acetyl-CoA into the ER Lumen Causes Neurodegeneration and Propensity to Infections, Inflammation, and Cancer. *The Journal of neuroscience : the official journal of the Society for Neuroscience.* 2014;34(20):6772-6789.

For Review Only

10. Peng Y, Kim MJ, Hullinger R, et al. Improved proteostasis in the secretory pathway rescues Alzheimer's disease in the mouse. *Brain : a journal of neurology.* 2016;139(Pt 3):937-952.

11. Hullinger R, Li M, Wang J, et al. Increased expression of AT-1/SLC33A1 causes an autistic-like phenotype in mice by affecting dendritic branching and spine formation. *J Exp Med.* 2016;213(7):1267-1284.

12. Peng Y, Shapiro SL, Banduseela VC, et al. Increased transport of acetyl-CoA into the endoplasmic reticulum causes a progeria-like phenotype. *Aging Cell.* 2018:e12820.

13. Sheehan BK, Orefice NS, Peng Y, Shapiro SL, Puglielli L. ATG9A regulates proteostasis through reticulophagy receptors FAM134B and SEC62 and folding chaperones CALR and HSPB1. *iScience.* 2021;24(4):102315.

14. Rigby MJ, Lawton AJ, Kaur G, et al. Endoplasmic reticulum acetyltransferases Atase1 and Atase2 differentially regulate reticulophagy, macroautophagy and cellular acetyl-CoA metabolism. *Commun Biol.* 2021;4(1):454.

15. Fernandez-Fuente G, Overmyer KA, Lawton AJ, et al. The citrate transporters SLC13A5 and SLC25A1 elicit different metabolic responses and phenotypes in the mouse. *Commun Biol.* 2023;6(1):926.

16. Fernandez-Fuente G, Rigby MJ, Puglielli L. Intracellular Citrate/acetyl-CoA flux and endoplasmic reticulum acetylation: Connectivity is the answer. *Mol Metab.* 2023;67:101653.

17. Jonas MC, Pehar M, Puglielli L. AT-1 is the ER membrane acetyl-CoA transporter and is essential for cell viability. *J Cell Sci.* 2010;123(Pt 19):3378-3388.

18. Ko MH, Puglielli L. Two Endoplasmic Reticulum (ER)/ER Golgi Intermediate Compartment-based Lysine Acetyltransferases Post-translationally Regulate BACE1 Levels. *J Biol Chem.* 2009;284(4):2482-2492.

19. Pehar M, Jonas MC, Hare TM, Puglielli L. SLC33A1/AT-1 protein regulates the induction of autophagy downstream of IRE1/XBP1 pathway. *J Biol Chem.* 2012;287(35):29921-29930.

20. Hullinger R, Li M, Wang J, et al. Increased expression of AT-1/SLC33A1 causes an autistic-like phenotype in mice by affecting dendritic branching and spine formation. *The Journal of experimental medicine.* 2016;213:1267-1284.

For Review Only

21. Murie M, Peng Y, Rigby MJ, et al. ATase inhibition rescues age-associated proteotoxicity of the secretory pathway. *Commun Biol.* 2022;5(1):173.

22. Kurth I, Pamminger T, Hennings JC, et al. Mutations in FAM134B, encoding a newly identified Golgi protein, cause severe sensory and autonomic neuropathy. *Nat Genet.* 2009;41(11):1179-1181.

23. Murphy SM, Davidson GL, Brandner S, Houlden H, Reilly MM. Mutation in FAM134B causing severe hereditary sensory neuropathy. *J Neurol Neurosurg Psychiatry.* 2012;83(1):119-120.

24. Almeida-Souza L, Goethals S, de Winter V, et al. Increased monomerization of mutant HSPB1 leads to protein hyperactivity in Charcot-Marie-Tooth neuropathy. *J Biol Chem.* 2010;285(17):12778-12786.

25. Capponi S, Geroldi A, Fossa P, et al. HSPB1 and HSPB8 in inherited neuropathies: study of an Italian cohort of dHMN and CMT2 patients. *J Peripher Nerv Syst.* 2011;16(4):287-294.

26. Evgrafov OV, Mersiyanova I, Irobi J, et al. Mutant small heat-shock protein 27 causes axonal Charcot-Marie-Tooth disease and distal hereditary motor neuropathy. *Nat Genet.* 2004;36(6):602-606.

27. Lin P, Li J, Liu Q, et al. A missense mutation in SLC33A1, which encodes the acetyl-CoA transporter, causes autosomal-dominant spastic paraplegia (SPG42). *Am J Hum Genet.* 2008;83(6):752-759.

28. De Pace R, Skirzewski M, Damme M, et al. Altered distribution of ATG9A and accumulation of axonal aggregates in neurons from a mouse model of AP-4 deficiency syndrome. *PLoS genetics.* 2018;14(4):e1007363.

29. Behne R, Teinert J, Wimmer M, et al. Adaptor protein complex 4 deficiency: a paradigm of childhood-onset hereditary spastic paraplegia caused by defective protein trafficking. *Hum Mol Genet.* 2020;29(2):320-334.

30. van Paassen BW, van der Kooi AJ, van Spaendonck-Zwarts KY, Verhamme C, Baas F, de Visser M. PMP22 related neuropathies: Charcot-Marie-Tooth disease type 1A and Hereditary Neuropathy with liability to Pressure Palsies. *Orphanet J Rare Dis.* 2014;9:38.

31. Pisciotta C, Shy ME. Hereditary neuropathy. *Handb Clin Neurol.* 2023;195:609-617.

For Review Only

32. Guyenet SJ, Furrer SA, Damian VM, Baughan TD, La Spada AR, Garden GA. A simple composite phenotype scoring system for evaluating mouse models of cerebellar ataxia. *J Vis Exp.* 2010(39).

33. Goebbels S, Oltrogge JH, Kemper R, et al. Elevated phosphatidylinositol 3,4,5-trisphosphate in glia triggers cell-autonomous membrane wrapping and myelination. *J Neurosci.* 2010;30(26):8953-8964.

34. Henry EW, Cowen JS, Sidman RL. Comparison of Trembler and Trembler-J mouse phenotypes: varying severity of peripheral hypomyelination. *Journal of neuropathology and experimental neurology.* 1983;42(6):688-706.

35. Suter U, Moskow JJ, Welcher AA, et al. A leucine-to-proline mutation in the putative first transmembrane domain of the 22-kDa peripheral myelin protein in the trembler-J mouse. *Proc Natl Acad Sci U S A.* 1992;89(10):4382-4386.

36. Suter U, Welcher AA, Ozcelik T, et al. Trembler mouse carries a point mutation in a myelin gene. *Nature.* 1992;356(6366):241-244.

37. Colby J, Nicholson R, Dickson KM, et al. PMP22 carrying the trembler or trembler-J mutation is intracellularly retained in myelinating Schwann cells. *Neurobiology of disease.* 2000;7(6 Pt B):561-573.

38. Dickson KM, Bergeron JJ, Shames I, et al. Association of calnexin with mutant peripheral myelin protein-22 ex vivo: a basis for "gain-of-function" ER diseases. *Proc Natl Acad Sci U S A.* 2002;99(15):9852-9857.

39. Verhamme C, King RH, ten Asbroek AL, et al. Myelin and axon pathology in a long-term study of PMP22-overexpressing mice. *Journal of neuropathology and experimental neurology.* 2011;70(5):386-398.

40. Marinko JT, Carter BD, Sanders CR. Direct relationship between increased expression and mistrafficking of the Charcot-Marie-Tooth-associated protein PMP22. *J Biol Chem.* 2020;295(34):11963-11970.

41. Fratta P, Ornaghi F, Dati G, et al. A nonsense mutation in myelin protein zero causes congenital hypomyelination neuropathy through altered P0 membrane targeting and gain of abnormal function. *Hum Mol Genet.* 2019;28(1):124-132.

For Review Only

42. Castillo-Mariqueo L, Gimenez-Llort L. Clasping, ledge-score coordination and early gait impairments as primary behavioural markers of functional impairment in Alzheimer's disease. *Behavioural brain research.* 2022;435:114054.

43. Li QF, Dong Y, Yang L, et al. Neurofilament light chain is a promising serum biomarker in spinocerebellar ataxia type 3. *Mol Neurodegener.* 2019;14(1):39.

44. Rigby MJ, Ding Y, Farrugia MA, et al. The endoplasmic reticulum acetyltransferases ATase1/NAT8B and ATase2/NAT8 are differentially regulated to adjust engagement of the secretory pathway. *J Neurochem.* 2020;154(4):404-423.

45. Ptak CP, Peterson TA, Hopkins JB, Ahern CA, Shy ME, Piper RC. Homomeric interactions of the MPZ Ig domain and their relation to Charcot-Marie-Tooth disease. *Brain : a journal of neurology.* 2023.

46. Bai Y, Wu X, Brennan KM, et al. Myelin protein zero mutations and the unfolded protein response in Charcot Marie Tooth disease type 1B. *Ann Clin Transl Neurol.* 2018;5(4):445-455.

For Review Only

Figure legends

Figure 1. ATase inhibition rescues the phenotypic severity score of Pmp22^{Tr-J} mice.
(A) Phenotypic severity score represented as a sum of ledge, gait and hindlimb clasping (n>5 animals/group). *P<0.05, **P<0.005 and $^{\#}$P<0.0005 via two-way ANOVA (genotype x age; F males statistic = 409.8 and F females statistic = 270.4 for genotype factor). Histogram (n=12 animals/group), $^{\#}$P<0.0005 via two-way ANOVA (genotype x age; F statistic = 1256 for genotype factor). **(B)** Ledge, gait, and hindlimb clasp severity score of males (upper panel) and female (lower panel) (n>5 animals/group). *P<0.05 and **P<0.005 via two-way ANOVA (genotype x age; F males statistic = 310.6 : 169.8 : 168.7 and F females statistic = 267.4 : 72.95 : 170.1 for ledge, gait and hindlimb clasp genotype factor respectively). **(C)** Representative image of WT, Pmp22^{Tr-J}, and Pmp22^{Tr-J} with C9 treatment animals at 5 months during the hindlimb clasp test.

Figure 2. ATase inhibition rescues spontaneous motor activity of Pmp22$^{Tr\text{-}J}$ mice.

(A) Total distance traveled. 2 month-old males (WT n=7; Pmp22$^{Tr\text{-}J}$ n=11; Pmp22$^{Tr\text{-}J}$ treated n=7), 2 month-old females (WT n=9; Pmp22$^{Tr\text{-}J}$ n=9; Pmp22$^{Tr\text{-}J}$ treated n=6), 5 month-old males (WT n=6; Pmp22$^{Tr\text{-}J}$ n=7; Pmp22$^{Tr\text{-}J}$ treated n=8), 5 month-old females (n=6 animals per group). *P<0.05, **P<0.005 and $^{\#}$P<0.0005 via two-way ANOVA (genotype x age; F males statistic = 56.91 and F females statistic = 16.07 for genotype factor)

(B) Rear/hind legs standing. 2 month-old males (WT n=7; Pmp22$^{Tr\text{-}J}$ n=9; Pmp22$^{Tr\text{-}J}$ treated n=7), 2 month-old females (WT n=9; Pmp22$^{Tr\text{-}J}$ n=9; Pmp22$^{Tr\text{-}J}$ treated n=6), 5 month-old males (WT n=6; Pmp22$^{Tr\text{-}J}$ n=6; Pmp22$^{Tr\text{-}J}$ treated n=7), 5 month-old females (WT n=6; Pmp22$^{Tr\text{-}J}$ n=5; Pmp22$^{Tr\text{-}J}$ treated n=6). *P<0.05, **P<0.005 and $^{\#}$P<0.0005 via two-way ANOVA (genotype x age; F males statistic = 72.25 and F females statistic = 18.79 for genotype factor)

Figure 3. ATase inhibition improves myelin morphology in the Pmp22$^{Tr\text{-}J}$ mice.

(A) Electron micrographs of the sciatic nerves at 5 months of Pmp22$^{Tr\text{-}J}$, Pmp22$^{Tr\text{-}J}$ treated, and WT littermates. **(B)** Percentage of non-myelinated axons (n=6 animals per group. Total axons per animal > 110). *P<0.05. *t*=2.32 via mean comparison using unpaired Student's *t*-test. **(C)** G-ratio value representation of sciatic nerves (n=5 animals/group. Total axons per animal > 25). $^{\#}$P<0.0005 via two-way ANOVA comparison (genotype x age; F statistic = 248.2 for genotype factor; F statistic = 1.19 for age factor). **(D)** G-ratio distribution according to axon diameter (n=5 animals per group. Total axons per animal > 25).

Figure 4. Phenotypic assessment of C3-PMP22 mice.

(A) Phenotypic severity score represented as a sum of ledge, gait and hindlimb clasping at different ages (n=7/group). **P<0.005 and $^{\#}$P<0.0005 via two-way ANOVA comparison (genotype x age; F statistic = 110.2 for genotype factor; F statistic = 33.99 for age factor).

(B) Open field assay. Total distance traveled. 2 months old (n=8/group). **(C)** Open field assay. Rears. 2 months old (n=8/group). **(D)** Hot plate time to reaction. 2 months old (n=8/group). **(E)** Grip strength normalized to body weight. 2 months old (n=8/group). **(F)** Time to fall from the

For Review Only

inverted screen. 2 months old (WT n=6; C3-PMP22 n=7; C3-PMP22 treated n=7). #P<0.0005 via one-way ANOVA (F statistic = 35.89). **(G)** Electron micrographs of the sciatic nerves at 5 months of C3-PMP22, C3-PMP22 treated, and WT littermates. **(H)** G-ratio value representation of sciatic nerves (n=5 animals/group. Total axons per animal > 35). *P<0.05 via one-way ANOVA comparison (F statistic = 5.2)

Figure 5. Phenotypic assessment of Mpz^{ttrr} mice.

(A) Phenotypic severity score represented as a sum of ledge, gait and hindlimb clasp at different ages (WT n=7; Mpz^{ttrr} n=7; Mpz^{ttrr} treated n=6). #P<0.0005 via two-way ANOVA comparison (genotype x age; F statistic = 769,4 for genotype factor; F statistic = 2.2 for age factor). **(B)** Open field assay. Total distance traveled. 2 months old (n=8 animals/group). *P<0.05 and **P<0.005 via one-way ANOVA comparison (F statistic = 6.56). **(C)** Open field assay. Rears. 2 months old (n=8 animals/group). #P<0.0005 via one-way ANOVA comparison (F statistic = 20.51). **(D)** Hot plate time to reaction. 2 months old (n=8 animals/group). #P<0.0005 via mean one-way ANOVA comparison (F statistic = 17.45) **(E)** Grip strength normalized to body weight. 2 months old (n=8 animals/group). #P<0.0005 via one-way ANOVA comparison (F statistic = 28.41). **(F)** Time to fall from the inverted screen. 2 months old (WT n=6; Mpz^{ttrr} n=7; Mpz^{ttrr} treated n=9). #P<0.0005 via one-way ANOVA (F statistic = 68.21). **(G)** Number of complete walks through the balance beam. (n=10 animals/group). #P<0.0005 via one-way ANOVA (F statistic = 106.8). **(H)** Electron micrographs of the sciatic nerves at 5 months of Mpz^{ttrr}, Mpz^{ttrr} treated, and WT littermates. **(I)** G-ratio value representation of sciatic nerves (n=5 animals/group. Total axons per animal >35. *P<0.05 and **P<0.005 via one-way ANOVA comparison (F statistic = 9.82).

For Review Only

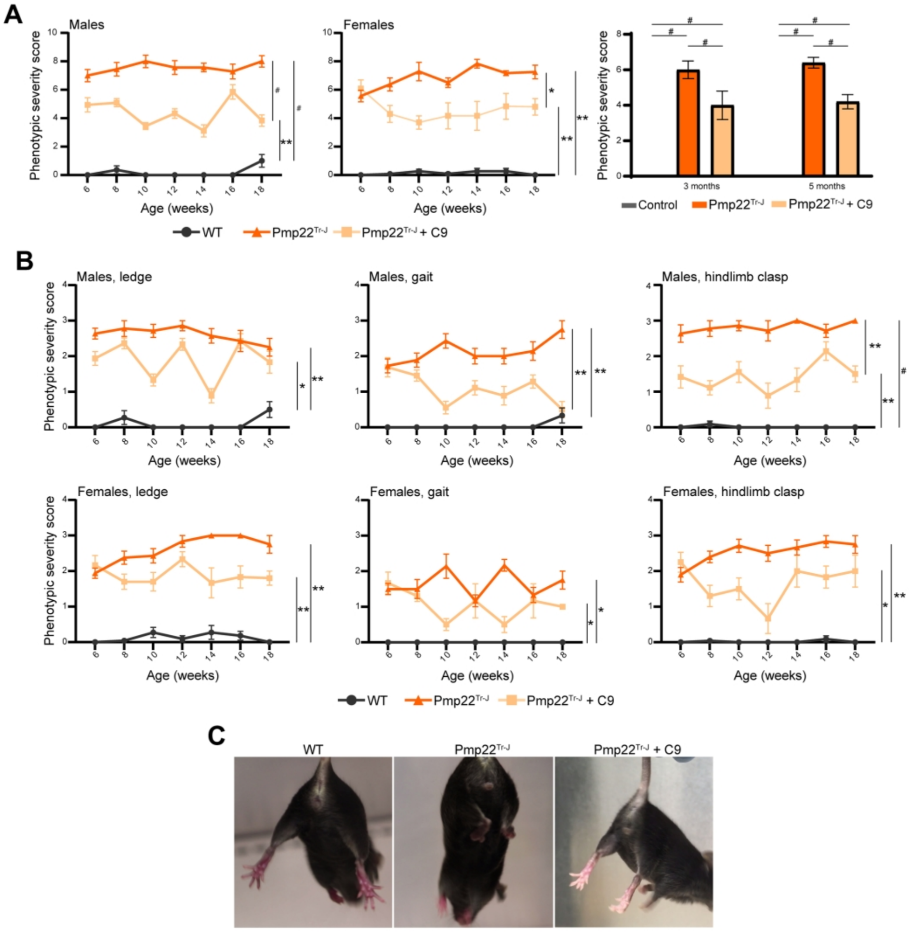

Figure 1

200x190mm (300 x 300 DPI)

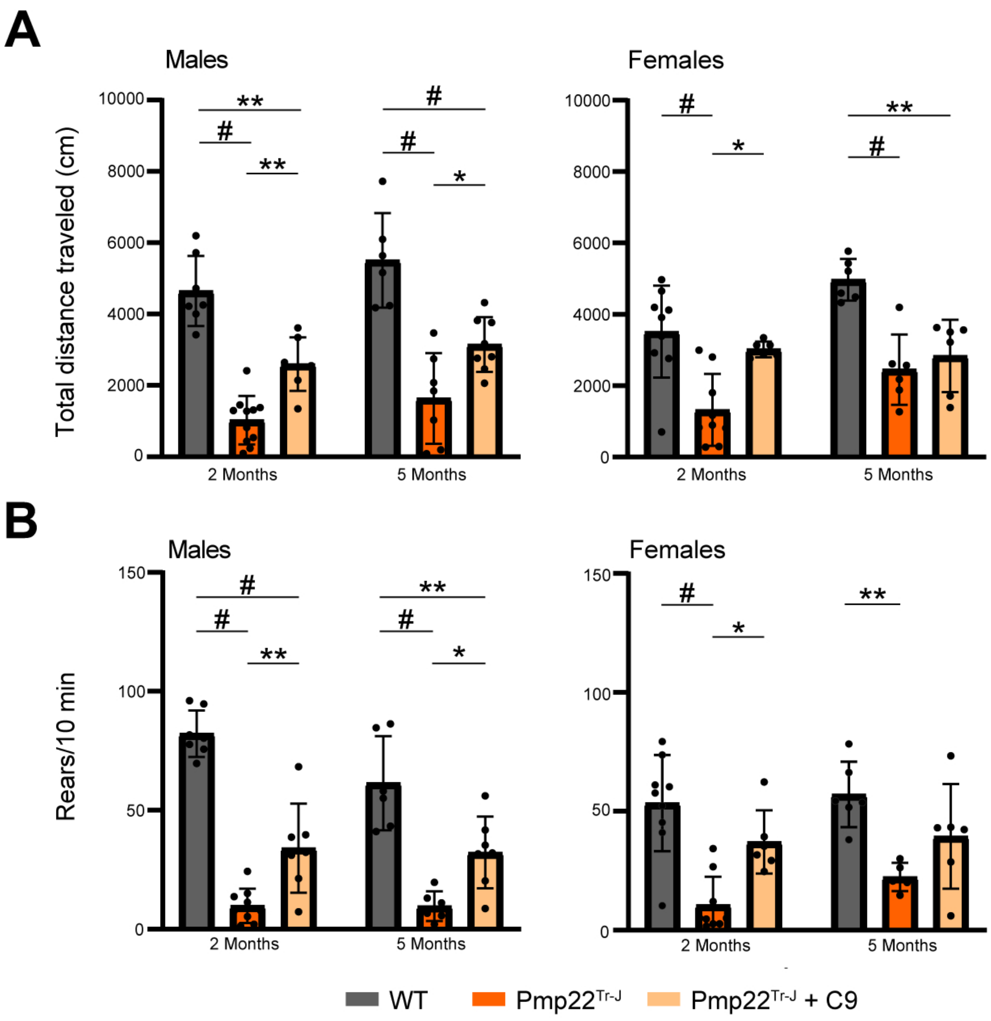

Figure 2

104x106mm (300 x 300 DPI)

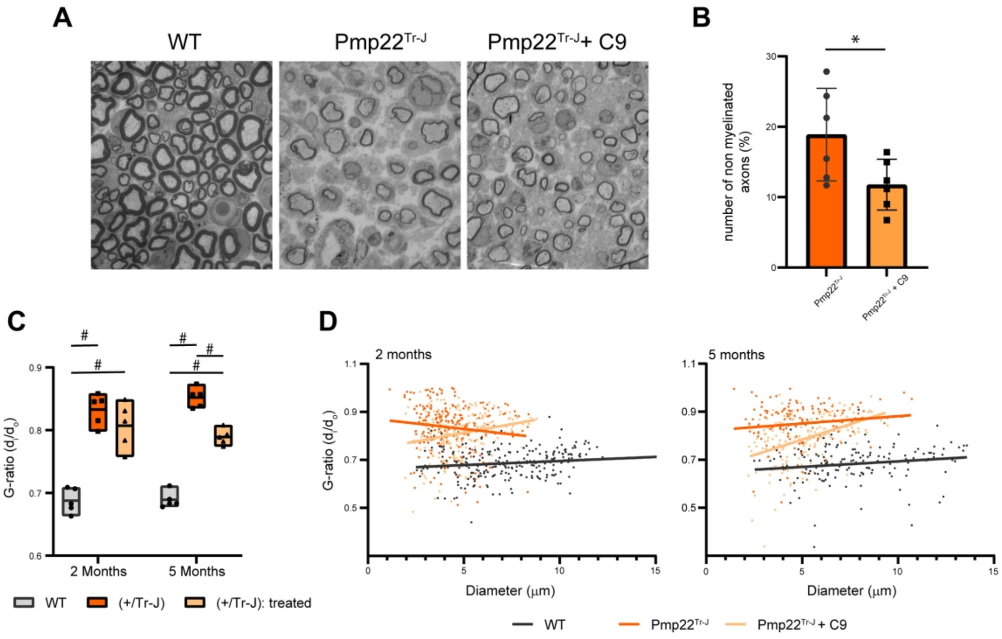

Figure 3

192x121mm (300 x 300 DPI)

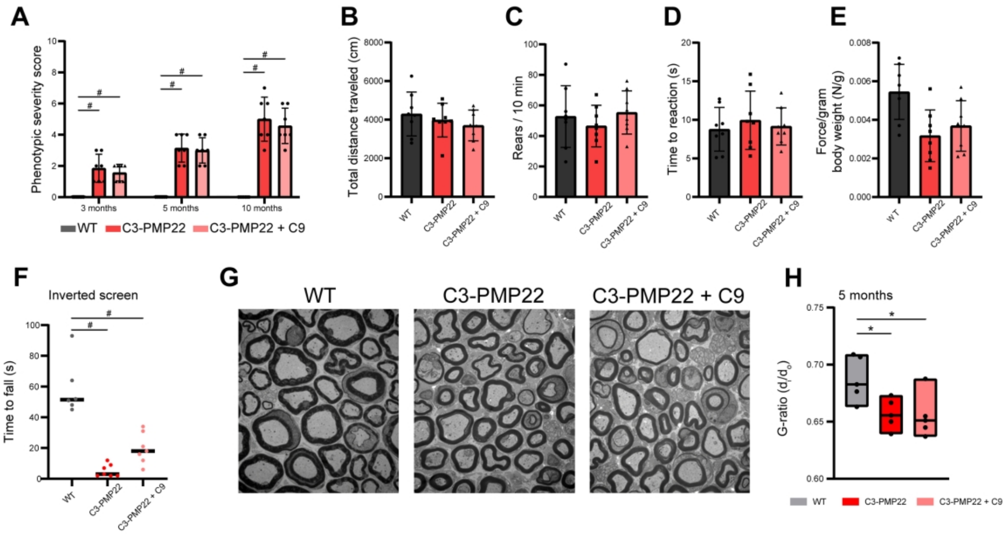

Figure 4

203x110mm (300 x 300 DPI)

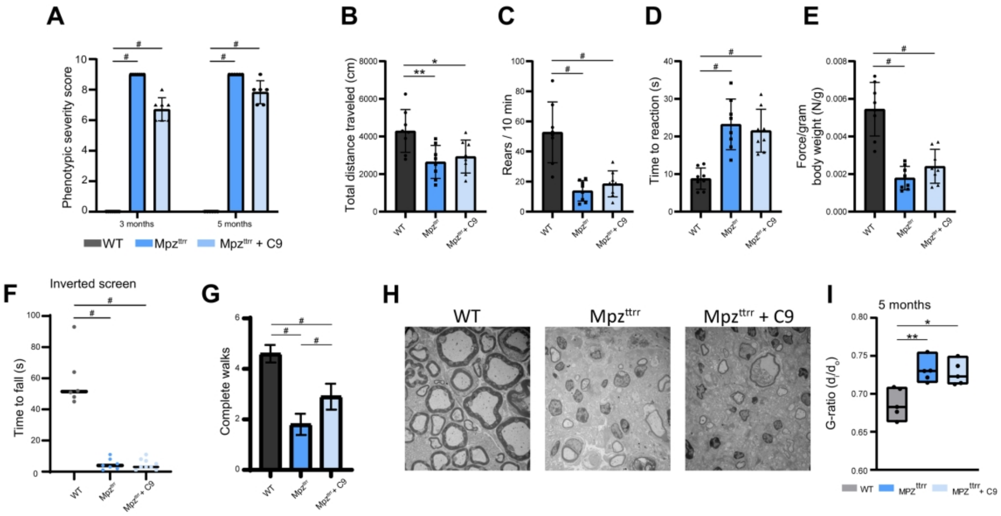

Figure 5

211x111mm (300 x 300 DPI)

SUPPLEMENTARY INFORMATION

Spatial selectivity of ATase inhibition in mouse models of Charcot-Marie-Tooth disease

Gonzalo Fernandez-Fuente[1,2,†], Mark A. Farrugia[1,2,†,#], Yajing Peng[1,2,$], Andrew Schneider[2], John Svaren[2,3], Luigi Puglielli[1,2,4*]

[†]**GF-F and MAF contributed equally to this paper**

[1]Department of Medicine, School of Medicine and Public Health, University of Wisconsin-Madison, Madison, WI 53705, USA

[2]Waisman Center, University of Wisconsin-Madison, Madison, WI 53705, USA

[3]Department of Comparative Biosciences, School of Veterinary Medicine, University of Wisconsin-Madison, Madison, WI 53706, USA

[4]Geriatric Research Education Clinical Center, Veterans Affairs Medical Center, Madison, WI 53705, USA

[#] Current affiliation: Department of Biochemistry & Molecular Biology, College of Natural Science, Michigan State University, East Lansing, MI, 48824

[$] Current affiliation: Wisconsin State Laboratory of Hygiene, University of Wisconsin-Madison, Madison, WI 53705, USA

For Review Only

Supplementary Table 1. Phenotypic Severity score.

Phenotypic Severity score

	0	1	2	3
**Ledge Wall**	No loss of balance, use of all paws, lowers itself into cage gracefully	Loss of balance observed	Poor hind limb use on ledge walk and/or drops into cage instead of lowering itself	Falls off ledge completely during walk, failure to clasp ledge or refusal to move
**Hindlimb clasp**	Both hind limbs splayed outward from midline for entire duration	Partial hind limb retraction of one hind limb toward midline for > 50% of time	Partial retraction of both hind limbs toward midline for > 50% of the time	Full retraction of both hind limbs toward midline for > 50% of time
**Gait**	Abdomen not touching the ground	Mild tremor or limp observed	Severe tremor/limp or paws pointing perpendicular to midline during walk	Refusal to move, abdomen drag-ging on floor during walk

Review Only

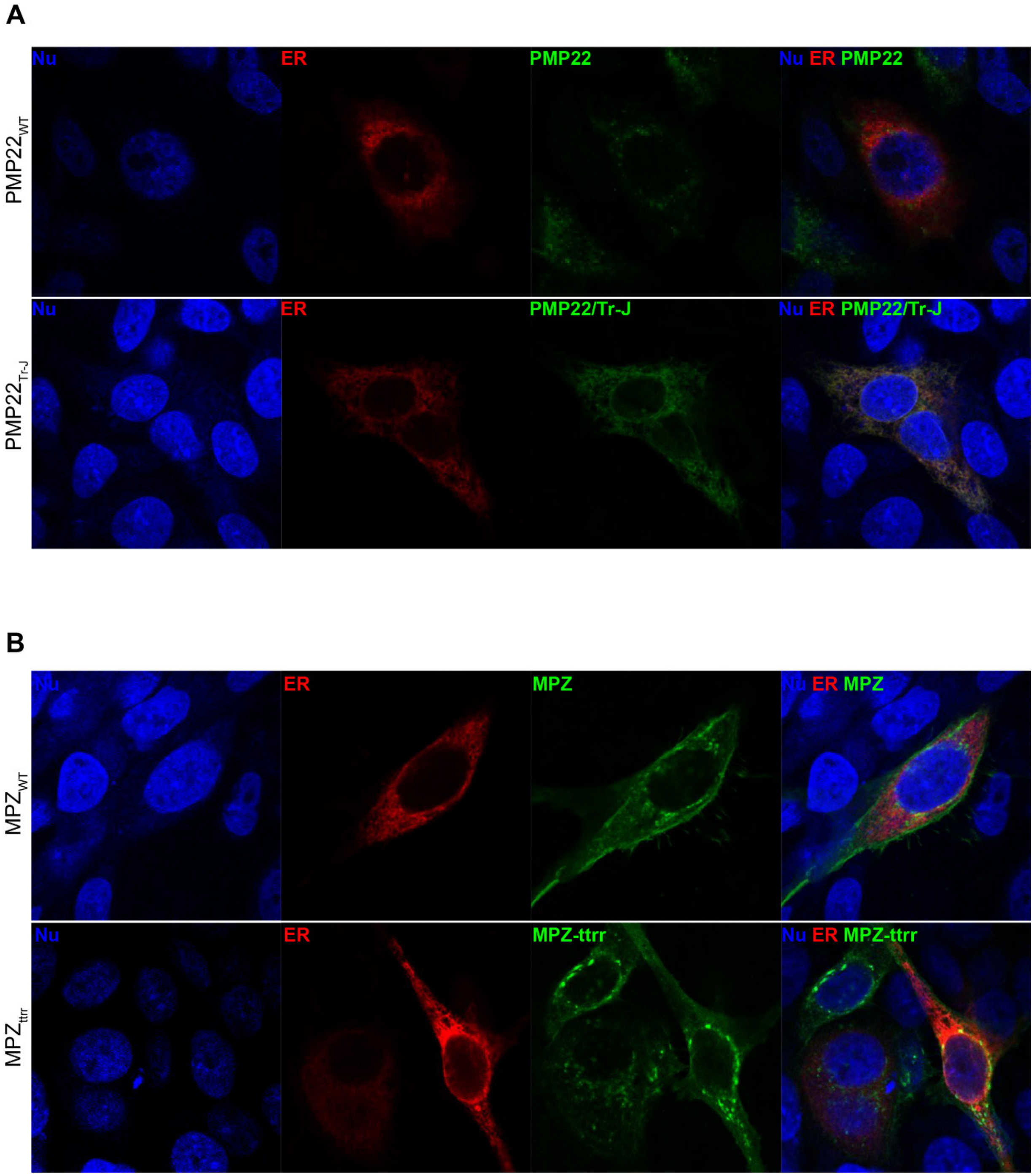

Supplementary Figure 1. $PMP22_{Tr\text{-}J}$, but not $PMP22_{WT}$, MPZ_{WT}, or MPZ_{ttrr}, forms aggregates in the lumen of the Endoplasmic Reticulum.

A) CHO cells were co-transfected with ER-mCherry and PMP22 (upper panel) or $PMP22_{Tr\text{-}J}$ (lower panel) plasmids.

B) CHO cells were co-transfected with ER-mCherry and MPZ (upper panel) or MPZ_{ttrr} (lower panel) plasmids.

For Review Only

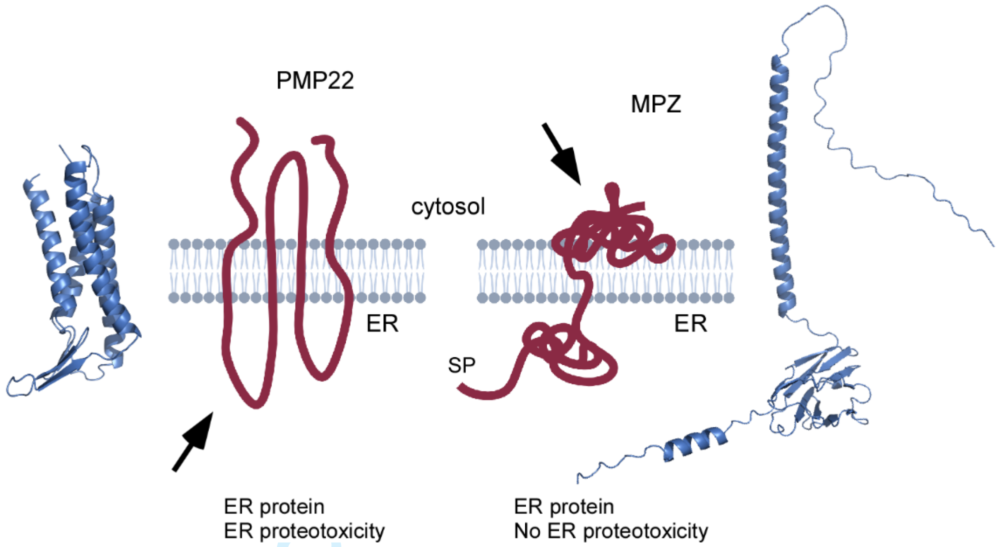

Supplementary Figure 2. Schematic view of the predicted topologies of PMP22 and MPZ with their Alphafold structures. Left: human PMP22, AF-Q01453. Right: human MPZ, AF-25189. Arrow points to the disease-associated mutations within the $PMP22_{Tr-J}$ and MPZ_{ttrr} mice. The cytosolic portion of the MPZ cartoon was partially adjusted to reflect Raasakka A, et al. Molecular structure and function of myelin protein P0 in membrane stacking. Sci Rep. 2019; 9:642. doi: 10.1038/s41598-018-37009-4.

For Review Only

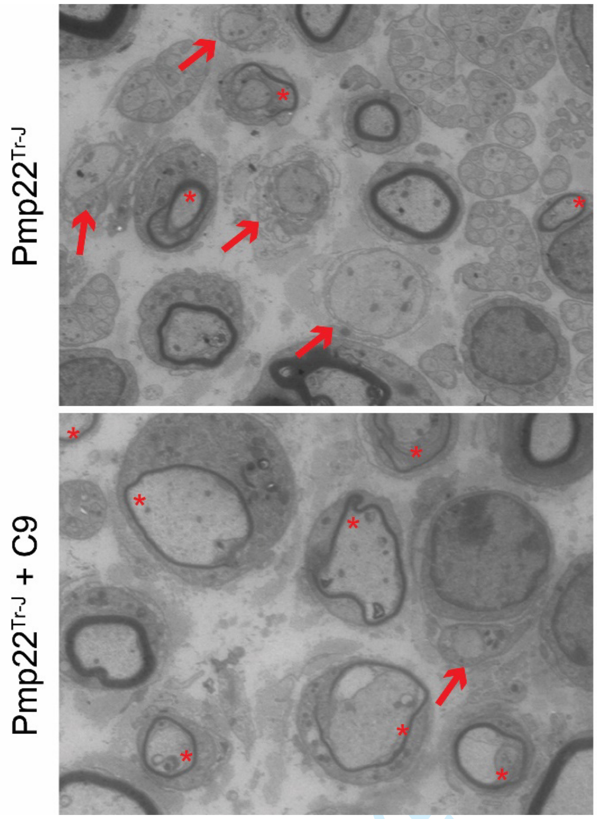

Supplementary Figure 3. ATase inhibtion improves myelin morphology in Pmp$^{22Tr\text{-}J}$ mice. Electron micrographs of the sciatic nerves at 5 months of Pmp22$^{Tr\text{-}J}$ and Pmp22$^{Tr\text{-}J}$ treated littermates. Arrows point to unmyelinated axons while asterisks point to “thin”-myelinated (probably re-myelinated) axons.
