# Supplementary material for: Spatial selectivity of ATase inhibition in mouse models of Charcot–Marie–Tooth disease
Source: Brain Commun. 2024 Jul 9;6(4):fcae232. doi: 10.1093/braincomms/fcae232 (PMC11258571; doi:10.1093/braincomms/fcae232)
Supplement: fcae232_Supplementary_Data [file fcae232_supplementary_data.zip › Supplementary_material.pdf]

SUPPLEMENTARY MATERIAL

Spatial selectivity of ATase inhibition in mouse models of Charcot-Marie-Tooth disease

Gonzalo Fernandez-Fuente[1,2,*], Mark A. Farrugia[1,2,*, †], Yajing Peng[1,2,‡], Andrew Schneider[2], John Svaren[2,3], Luigi Puglielli[1,2,4**]

[*]Gonzalo Fernandez-Fuente and Mark Farrugia contributed equally to this work.

1 Department of Medicine, School of Medicine and Public Health, University of Wisconsin-Madison, Madison, WI 53705, USA

2 Waisman Center, University of Wisconsin-Madison, Madison, WI 53705, USA

3 Department of Comparative Biosciences, School of Veterinary Medicine, University of Wisconsin-Madison, Madison, WI 53706, USA

4 Geriatric Research Education Clinical Center, Veterans Affairs Medical Center, Madison, WI 53705, USA

[†] Present address: Department of Biochemistry & Molecular Biology, College of Natural Science, Michigan State University, East Lansing, MI 48824, USA

[‡] Present address: Wisconsin State Laboratory of Hygiene, University of Wisconsin-Madison, Madison, WI 53705, USA

Supplementary Table 1. Phenotypic Severity score.

Phenotypic Severity score	0	1	2	3
**Ledge Wall**	No loss of balance, use of all paws, lowers itself into cage gracefully	Loss of balance observed	Poor hind limb use on ledge walk and/or drops into cage instead of lowering itself	Falls off ledge completely during walk, failure to clasp ledge or refusal to move
**Hindlimb clasp**	Both hind limbs splayed outward from midline for entire duration	Partial hind limb retraction of one hind limb toward midline for > 50% of time	Partial retraction of both hind limbs toward midline for > 50% of the time	Full retraction of both hind limbs toward midline for > 50% of time
**Gait**	Abdomen not touching the ground	Mild tremor or limp observed	Severe tremor/limp or paws pointing perpendicular to midline during walk	Refusal to move, abdomen dragging on floor during walk

A

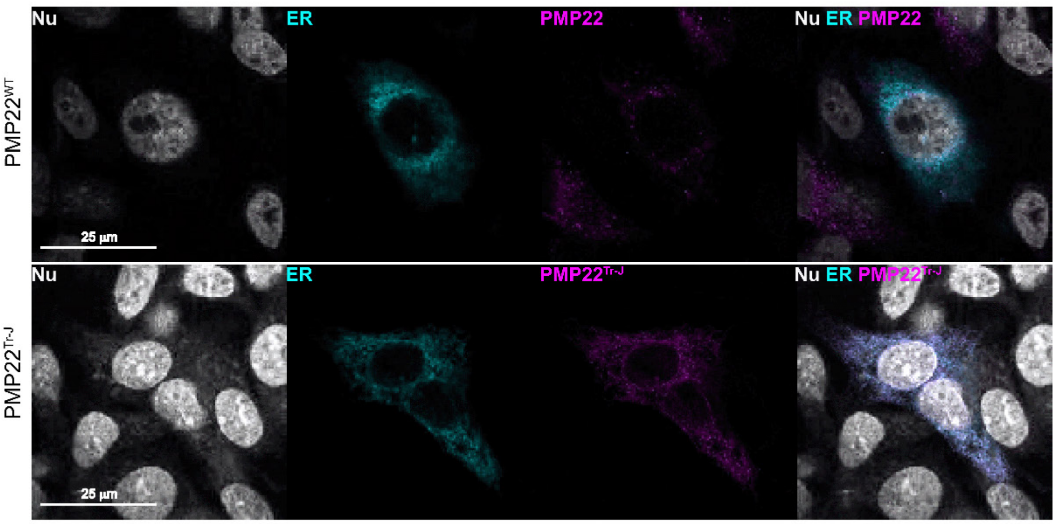
Nu
ER
PMP22
Nu ER PMP22
PMP22WT
25 µm
Nu
ER
PMP22Tr-J
Nu ER PMP22Tr-J
PMP22Tr-J
25 µm

B

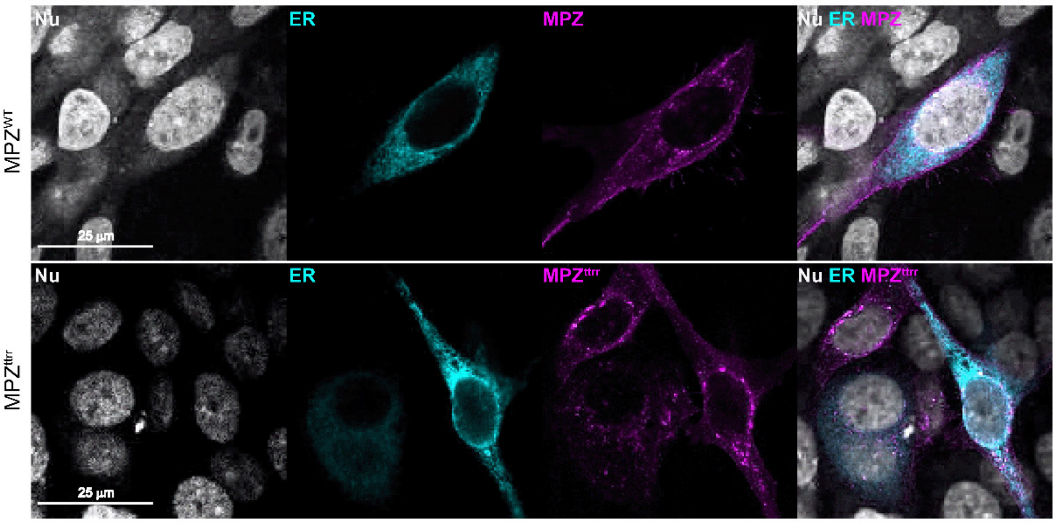
Nu
ER
MPZ
Nu ER MPZ
MPZWT
25 µm
Nu
ER
MPZttrr
Nu ER MPZttrr
MPZttrr
25 µm

C

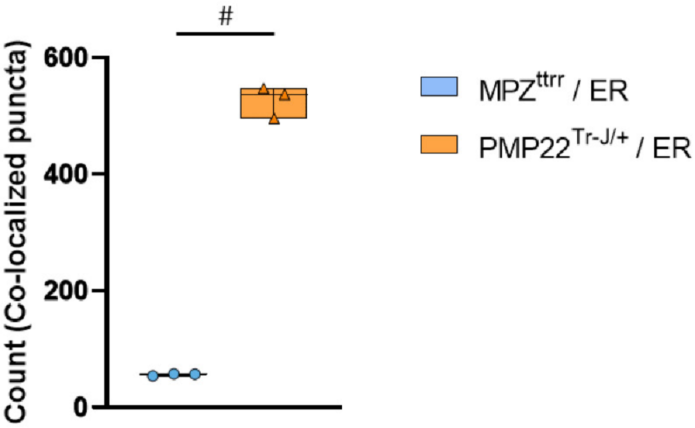
#
Count (Co-localized puncta)
0
200
400
600
MPZttrr / ER
PMP22Tr-J/+ / ER

Supplementary Figure 1. $PMP22_{Tr\text{-}J}$, but not $PMP22_{WT}$, MPZ_{WT}, or MPZ_{ttrr}, forms aggregates in the lumen of the Endoplasmic Reticulum.

A) CHO cells were co-transfected with ER-mCherry and PMP22 (upper panel) or $PMP22_{Tr\text{-}J}$ (lower panel) plasmids.

B) CHO cells were co-transfected with ER-mCherry and MPZ (upper panel) or MPZ_{ttrr} (lower panel) plasmids.

C) Puncta quantification of $PMP22_{Tr\text{-}J}$ and MPZ_{ttrr} with ER colocalization (n = 3 culture replicates, 100 cells per sample). $^{\#}$ P <0.0005 via mean comparison using unpaired Student's *t*-test (t=29.99).

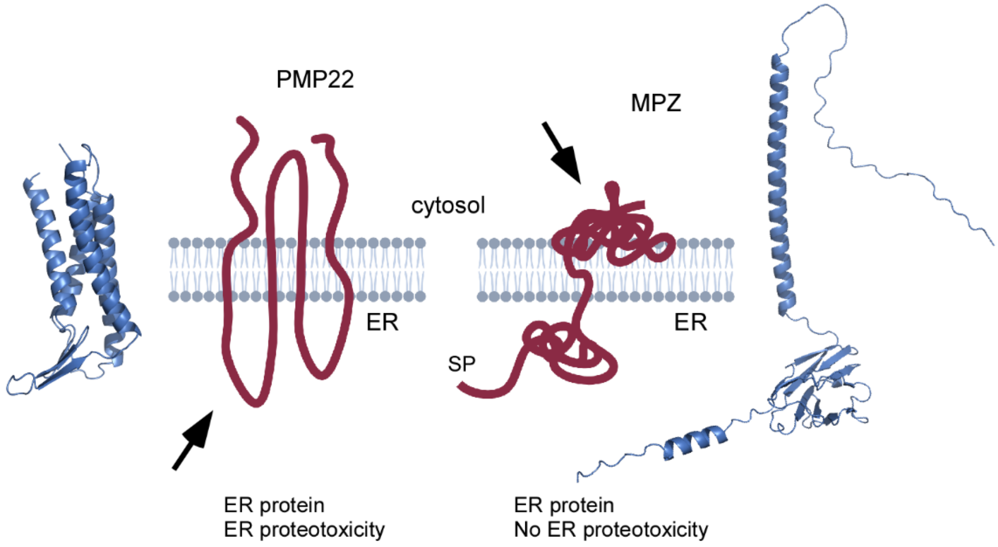

Supplementary Figure 2. Schematic view of the predicted topologies of PMP22 and MPZ with their Alphafold structures. Left: human PMP22, AF-Q01453. Right: human MPZ, AF-25189. Arrow points to the disease-associated mutations within the $PMP22_{Tr\text{-}J}$ and MPZ_{ttrr} mice. The cytosolic portion of the MPZ cartoon was partially adjusted to reflect Raasakka A, et al. Molecular structure and function of myelin protein P0 in membrane stacking. Sci Rep. 2019; 9:642. doi: 10.1038/s41598-018-37009-4. ER : endoplasmic reticulum; MPZ : myelin protein zero; PMP22 : peripheral myelin protein 22; SP : signal peptide.

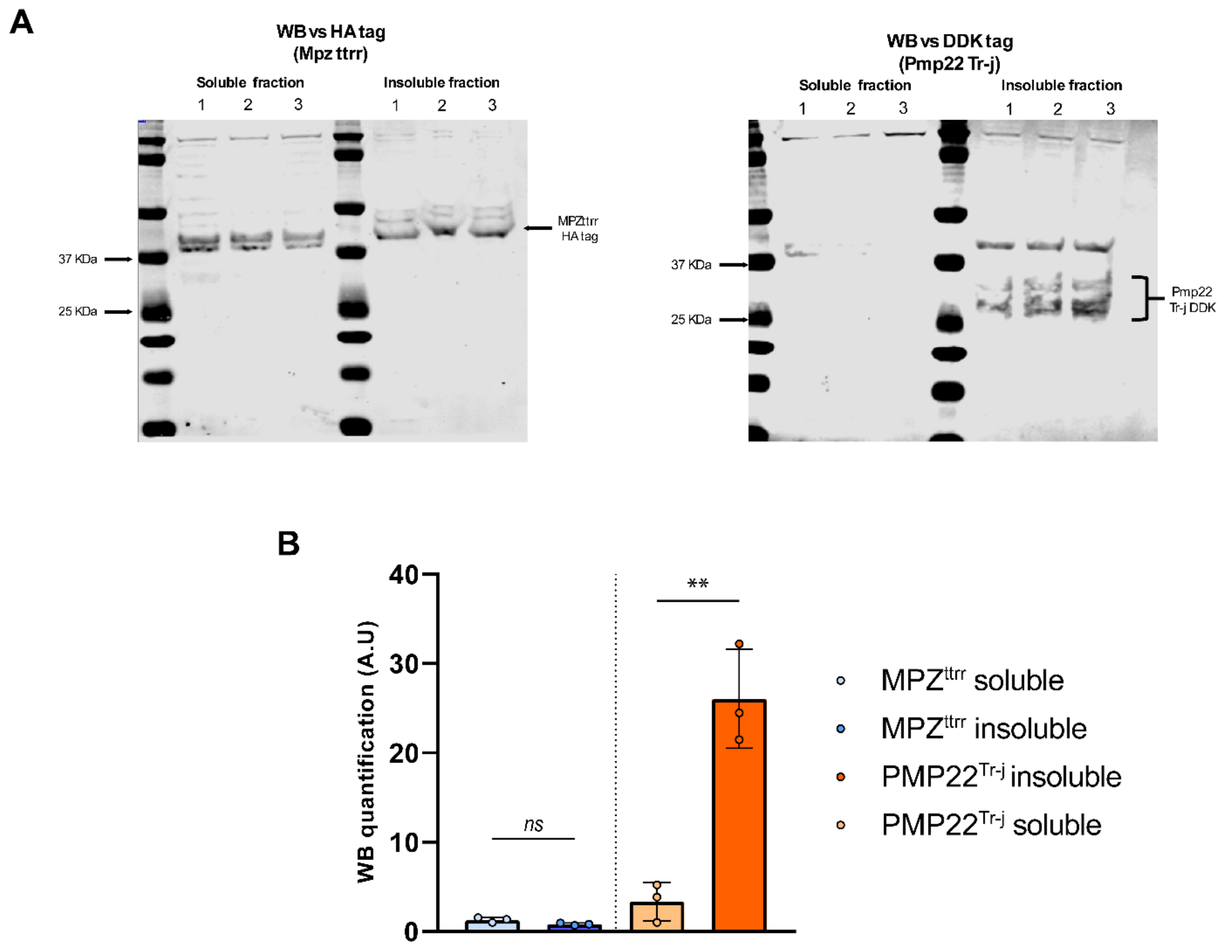

Supplementary Figure 3. Differential pro-aggregating properties of $PMP22_{Tr-J}$ and MPZ_{ttrr} mutants.

A) Western blot showing the migration profile of MPZ_{ttrr} and $PMP22_{Tr-J}$.

B) Quantification of changes showing the levels of soluble (Triton™ X-100) and insoluble/aggregated (SDS) species. Relative ratios are shown (n=3 culture replicates).

** P <0.005 via mean comparison using unpaired Student's *t*-test (MPZ t=2.753; PMP22 t=6.623).

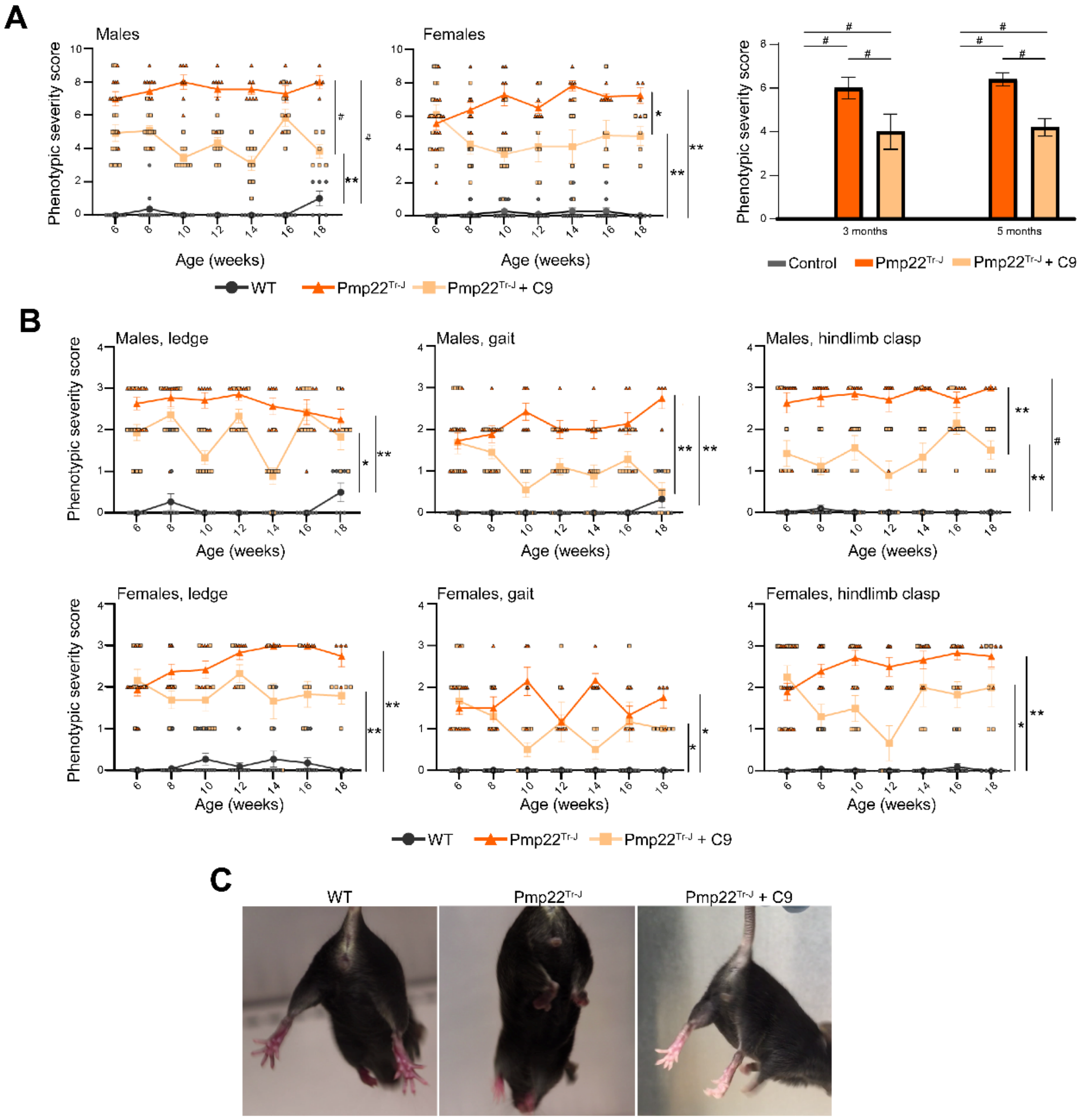

Supplementary Figure 4. ATase inhibition rescues the phenotypic severity score of Pmp22$^{Tr\text{-}J}$ mice. For the legend, see Figure 1.

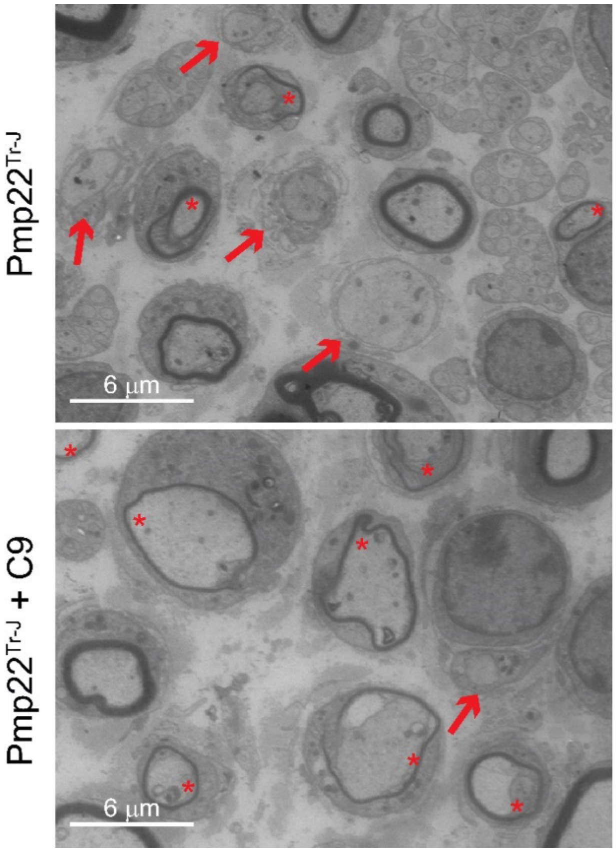

Supplementary Figure 5. ATase inhibition improves myelin morphology in Pmp$^{22Tr\text{-}J}$ mice. Electron micrographs of the sciatic nerves at 5 months of Pmp22$^{Tr\text{-}J}$ and Pmp22$^{Tr\text{-}J}$ treated littermates. Arrows point to unmyelinated axons while asterisks point to “thin”-myelinated (probably re-myelinated) axons.
