# Supplementary material for: Spatial selectivity of ATase inhibition in mouse models of Charcot–Marie–Tooth disease
Source: Brain Commun. 2024 Jul 9;6(4):fcae232. doi: 10.1093/braincomms/fcae232 (PMC11258571; doi:10.1093/braincomms/fcae232)
Supplement: fcae232_Supplementary_Data [file Original Submission - Author's Response.pdf › Original Submission - Author's Response.pdf]

POINT-BY-POINT RESPONSE TO REVIEWERS

We thank the Editors and Reviewers for their positive comments. Below are specific responses.

Reviewer: 1

We thank the Reviewer for the positive comments.

Reviewer: 2

...The image provided in Fig. S1 is not convincing and not quantified. In fact, it appears to me that the MPZ mutant does, in fact, aggregate in the ER. Therefore, it is incumbent upon the authors to provide convincing, quantitative data that this mutation does not form aggregates in the ER.

Response: We have added quantitative data to Supplementary Figure 1. We have also expressed $PMP22_{Tr\text{-}J}$ and MPZ_{ttrr} mutant in cultured cells and separated both soluble and non-soluble (aggregated) versions of the proteins. The results are shown in Supplementary Figure 3. In contrast to MPZ_{ttrr}, which has the distribution profile of a typical misfolded protein (ratio 1:1, soluble:insoluble), $PMP22_{Tr\text{-}J}$ is almost exclusively found the aggregated form (ratio 1:25, soluble/insoluble).

Comments from the Scientific Editorial team:

-*Thank you for providing an Abbreviated Summary. Please spell out the abbreviations included in the abbreviated summary.*

Response: Done as requested.

-*Thank you for providing a graphical abstract. Please also spell out the abbreviations included in the graphical abstract.*

Response: Done as requested.

-*Data availability: Please provide both data and the codes generated and used in this work within the supplementary material or through a repository, whose link has to be made available within the data availability statement.*

Response: The raw data are included as an Excel file (see the *Data availability* section). No codes were generated as part of this study.

-Figure 1: For each statistical analysis part of a figure, please report the exact sample size (N=), and if the number is <10 show the single data-points.

Response: The graph is generated with single-value points (see Materials and Methods and Supplementary Table 1). As such, the single data-point format might be difficult to visualize. We would suggest leaving the current Figure 1 and adding the single-point format as Supplementary Figure (see Supplementary Figure 4).

-Figures 3A, 4G, 5H, Supplementary figures 1 and 3: Please add a scale bar to these figures.

Response: Done as requested.

-Figures 4B-E: For each statistical analysis part of a figure, please report in the figure legend the statistical test used and relevant test statistic values alongside p values (for example F or t values for ANOVAs and t-tests).

Response: Done as requested.

-Supplementary figure 1: Please revisit your colour choice in this figure as the green-red combination is not colour-blind friendly. You may consider replacing the red channel with magenta, and green channel with cyan to make the figure colour-blind friendly.

Response: Done as requested.

-Supplementary Figure 2: Please ensure all abbreviations used in the figure and figure legend are defined within the figure legend so that they may be easily interpreted as stand-alone figures.

Response: Done as requested.

-At the revision stage, if you plan to edit the current figures and tables, or to make new figures and tables, please make sure that these items fit a typeset journal page (A4).

Response: Done as requested.
