# Supplementary material for: Spatial selectivity of ATase inhibition in mouse models of Charcot–Marie–Tooth disease
Source: Brain Commun. 2024 Jul 9;6(4):fcae232. doi: 10.1093/braincomms/fcae232 (PMC11258571; doi:10.1093/braincomms/fcae232)
Supplement: fcae232_Supplementary_Data [file Original Submission - Decision Letter.pdf › Original Submission - Decision Letter.pdf]

Original Submission - Decision Letter

19-Mar-2024

Dear Dr. Puglielli,

Thank you for your recent submission BRAINCOM-2024-121 entitled "Spatial selectivity of ATase inhibition in mouse models of Charcot-Marie-Tooth disease" to Brain Communications.

Your manuscript has been reviewed, and while the reviewers had largely positive comments, they have some constructive critiques that I think you could address in a minor revision of the manuscript. Therefore, I invite you to respond to the reviewer(s)' comments and revise your manuscript. The reviewer(s)' comments are included at the bottom of this message.

In addition to the reviewers' comments, please also address our Scientific Editor's comments added below to comply with our requirements for presentation of scientific data at Brain Communications.

Please format your revised manuscript according to our Instructions to Authors https://academic.oup.com/braincomms/pages/General_Instructions .
A summary of key points is attached to help with this. Please note that there can be substantial delays to publication if you don't resubmit in line with our requirements.

To revise your manuscript, click the link below or log into https://mc.manuscriptcentral.com/braincom and enter your Author Centre, where you will find your manuscript title listed under "Manuscripts with Decisions." Under "Actions," click on "Create a Revision." Your manuscript number will be appended to denote a revision.

*** PLEASE NOTE: This is a two-step process. After clicking on the link, you will be directed to a webpage to confirm. ***

https://mc.manuscriptcentral.com/braincom?URL_MASK=017b0c7978794816a01687b0d12ea643

Please upload a revised manuscript with the changes you have made shown either with "track changes" or in coloured text.

When submitting your revised manuscript, you will be asked to respond to the reviewer(s) comments. Please be as specific as possible in your response to all of the comments.

The editorial team at Brain Communications also aims to increase transparency in scientific publishing and to demonstrate the value that peer review brings to the scientific process. Please therefore consider whether you are willing to have reviewer comments on your paper and your response published as supplementary data with the paper if it is accepted. This is entirely optional and will only be done if both the authors and reviewers agree. When submitting your revision you will have the opportunity to update the answer you entered previously.

IMPORTANT: Your original files are available to you when you upload your revised manuscript. Please delete any redundant files before completing the submission.

Because we are trying to facilitate timely publication of manuscripts submitted to Brain Communications, we would appreciate your revised manuscript as soon as possible. If it is not possible for you to submit your revision within 3 months, the option to submit a revision will expire, and we will consider your revised paper as a new submission. However, if you need a little more time, please get in touch and we can extend this deadline for you.

Once again, thank you for submitting your manuscript to Brain Communications and I look forward to receiving your revision.

Best wishes

Professor Tara Spires-Jones
Founding Editor, Brain Communications

Reviewer(s)' Comments to Author:

Reviewer: 1 (Michael Shy)

Comments to the Author
Fernandez-Fuente et al report on Spatial selectivity of ATase inhibition of three mouse models of demyelinating CMT. They note that ATase1 and 2 have an essential role in the ER role of correctly folding polypeptide and removal of toxic protein aggregates in the ER such as occur in these mice and likely patients with similar mutations. They show that inhibiting ATases activity targets misfolding/pro-aggregating events occurring in the ER and establish the ATases as targets that could be addressed in manipulating disease-causing proteotoxic states. The paper is interesting, well written with solid data. The manuscript has been reviewed previously at Brain. To this reviewer the authors have responded adequately to all of the concerns raised in the prior review. I have no further concerns.

Reviewer: 2 (Bruce Carter)

Comments to the Author
In this manuscript, Fernandez-Fuente et al report on the use of a selective inhibitor of a lysine acetyl transferase (ATase), involved in ER specific autophagy, to improve the pathology in the Trembler-J (TrJ) model of CMT1E. The inhibitor significantly improved the overall neuropathy in the TrJ mice, both at the behavioral and histological levels. These authors have previously published on the mechanisms of action of their inhibitor (compound 9) and the focus of this manuscript was to assess the specificity of the effects for TrJ vs 2 other CMT models, the C3 mouse, which mimics CMT1A, and MPZttrr, which mimics a form of CMT1B. The study is well controlled and they assessed a variety of end points. It will certainly be of high interest to the community, especially given the clinical implications. The manuscript was previously reviewed at Brain and they have addressed most of the comments of the reviewer. However, one issue that has not been adequately responded to is the point about the formation of protein aggregates in the ER. The authors' response was to point out that the formation of ER aggregates by the TrJ mutant PMP22 is well described in the literature, which is true. However, the MPZttrr mutant has not been investigated. Many MPZ mutation DO form aggregates and activate UPR pathways in the ER ( ). Unfortunately, this "totterer" mouse does not seem to be described in the literature, at least as far as I can find. The link provided by JAX is just some type of preprint superficially characterizing the mouse and does not include any information about protein aggregation. The point of their manuscript is that the ATase inhibitor selectively improves the pathology when proteins aggregate in the ER, due to its ability to promote ER-phagy. The manuscript describes beneficial effects of compound 9 in the TrJ, but not the C3 or MPZttrr mouse. However, if the MPZttrr mutant does aggregate in the ER, like many MPZ mutants, then their conclusion that compound 9 is effective for any protein with ER aggregates may not hold, although Fig. 5 does show some improvements with the treatment. The image provided in Fig. S1 is not convincing and not quantified. In fact, it appears to me that the MPZ mutant does, in fact, aggregate in the ER. Therefore, it is incumbent upon the authors to provide convincing, quantitative data that this mutation does not form aggregates in the ER.

Comments from the Scientific Editorial team:

-Thank you for providing an Abbreviated Summary. Please spell out the abbreviations included in the abbreviated summary.

-Thank you for providing a graphical abstract. Please also spell out the abbreviations included in the graphical abstract.

-Data availability: Please provide both data and the codes generated and used in this work within the supplementary material or through a repository, whose link has to be made available within the data availability statement.

-Figure 1: For each statistical analysis part of a figure, please report the exact sample size (N=), and if the number is <10 show the single data-points.

-Figures 3A, 4G, 5H, Supplementary figures 1 and 3: Please add a scale bar to these figures.

-Figures 4B-E: For each statistical analysis part of a figure, please report in the figure legend the statistical test used and relevant test statistic values alongside p values (for example F or t values for ANOVAs and t-tests).

-Supplementary figure 1: Please revisit your colour choice in this figure as the green-red combination is not colour-blind friendly. You may consider replacing the red channel with magenta, and green channel with cyan to make the figure colour-blind friendly.

-Supplementary Figure 2: Please ensure all abbreviations used in the figure and figure legend are defined within the figure legend so that they may be easily interpreted as stand-alone figures.

-At the revision stage, if you plan to edit the current figures and tables, or to make new figures and tables, please make sure that these items fit a typeset journal page (A4).
