# Supplementary material for: Spatial selectivity of ATase inhibition in mouse models of Charcot–Marie–Tooth disease
Source: Brain Commun. 2024 Jul 9;6(4):fcae232. doi: 10.1093/braincomms/fcae232 (PMC11258571; doi:10.1093/braincomms/fcae232)
Supplement: fcae232_Supplementary_Data [file Revision 1 - Author's Response.pdf › Revision 1 - Author's Response.pdf]

POINT-BY-POINT RESPONSE TO THE SCIENTIFIC EDITOR

All changes are highlighted in the manuscript.

- *there is an issue with the ithenticate report (attached) related to the methods section. We cannot accept having portions of text completely copied and pasted from other articles. Please, edit this paragraph or replace the paragraph with the following wording: "the 'specified protocol' was performed accordingly to 'PAPER CITATION'.*

Response: Done as requested.

- *Fig3d: please report in the figure legend the relevant test statistic values alongside p values (for example F or t values for ANOVAs and t-tests).*

Response: We have already communicated on this on February 22, 2024 and it was approved during formal review. Specifically:

Panels 3C and 3D reflect the same data. The statistical analysis is associated with panel C using individual mice as the experimental unit (with ANOVA), as previously requested. Panel D simply displays the g-ratio distribution for each group without statistics.

We already argued (approved during formal review) that, even in the absence of statistical analysis, the distribution profile of the axonal g-ratio should be shown to the reader. In other words, we have not performed any statistical analysis using individual axons as the experimental unit and we do not make any statement about the axonal data. We simply show the data.

Please, note that this is in line with the current literature (selected examples: Biomedicines. 2023 Dec 17;11(12):3334. doi: 10.3390/biomedicines11123334. PMID: 38137555; PMCID: PMC10741921. Biochem Biophys Res Commun. 2022 Mar 15;597:1-7. doi: 10.1016/j.bbrc.2022.01.098. Epub 2022 Jan 29. PMID: 35121177. J Neurol Sci. 2015 Nov 15;358(1-2):213-20. doi: 10.1016/j.jns.2015.08.1539. Epub 2015 Aug 29. PMID: 26349404. EBioMedicine. 2024 Feb;100:104982. doi: 10.1016/j.ebiom.2024.104982. Epub 2024 Feb 1. PMID: 38306899; PMCID: PMC10850420. Front Neuroanat. 2023 Nov 22;17:1260186. doi: 10.3389/fnana.2023.1260186. PMID: 38074449; PMCID: PMC10702553. PLoS Comput Biol. 2023 Nov 17;19(11):e1010845. doi: 10.1371/journal.pcbi.1010845. PMID: 37976310; PMCID: PMC10691719. Acta Neuropathol Commun. 2023 Jun 20;11(1):100. doi: 10.1186/s40478-023-01598-7. PMID: 37340488; PMCID: PMC10283269. Brain. 2023 Aug 1;146(8):3331-3346. doi: 10.1093/brain/awad057. PMID: 37068912; PMCID: PMC10393406. Front Immunol. 2022 Sep 20;13:987771. doi: 10.3389/fimmu.2022.987771. PMID: 36203577; PMCID: PMC9531234. PLoS One. 2021 Jul 7;16(7):e0248323. doi: 10.1371/journal.pone.0248323. PMID: 34234376; PMCID: PMC8263263. eNeuro. 2021 Jul 21;8(4):ENEURO.0558-20.2021. doi: 10.1523/ENEURO.0558-20.2021. PMID: 34193510; PMCID: PMC8298095).

Some journals require the relative equations to accompany this type of graph, while some do not. Since we did not perform any statistical analysis of panel 3D we have removed the equations. However, if you wish, we can add them back.

- *Fig1c: please, clarify if each data point represents a biological replicate, also, please since the number is <10 show the single data-points. Please note our policy on pseudoreplication, we do not accept analyses where individual cells are the experimental unit when there is likely biological variability between animals, culture replicates, etc. Please see instructions for authors for more details. For this type of data, we require either that a mean/median be taken per animal/culture replicate followed by statistical analyses or that more sophisticated analyses are performed such as mixed effects modelling which can account for random variables*.

Response: This comment is for Supplementary Figure 1C (not Figure 1C). Done as requested.

- *Fig3b: please make sure to report in the figure legend, where missing, the test used and relevant test statistic values alongside p values (for example F or t values for ANOVAs and t-tests). Also, please, provide details of the experimental unit for each group analysed in these plots, and if the number is <10 show the single data-points. Please note: We do not accept analyses figure here individual measurements are the experimental unit when there is likely to be biological variability between animals, culture replicates, etc. For this type of data, we require either that a mean or median is taken per animal/culture replicate followed by statistical analyses, or that more sophisticated analyses are performed such as mixed effects modelling which can account for random effects.*

Response: This comment is for Supplementary Figure 3B (not Figure 3B). Done as requested.
