# Supplementary material for: Spatial selectivity of ATase inhibition in mouse models of Charcot–Marie–Tooth disease
Source: Brain Commun. 2024 Jul 9;6(4):fcae232. doi: 10.1093/braincomms/fcae232 (PMC11258571; doi:10.1093/braincomms/fcae232)
Supplement: fcae232_Supplementary_Data [file Revision 1 - Decision Letter.pdf › Revision 1 - Decision Letter.pdf]

Revision 1 - Decision Letter

28-May-2024

Dear Dr. Puglielli,

Thank you for your recent submission BRAINCOM-2024-121.R1
entitled "Spatial selectivity of ATase inhibition in mouse models of Charcot-Marie-Tooth disease" to Brain Communications.

I am pleased to let you know that we would like to provisionally accept your manuscript entitled "Spatial selectivity of ATase inhibition in mouse models of Charcot-Marie-Tooth disease" for publication in Brain Communications. There are still some minor points for you to address before final acceptance, but further peer-review will not be necessary.
Please would you respond to the comments shown below from reviewers and/or our Scientific Editor (regarding our requirements for presentation of scientific data at Brain Communications) and submit a revised version.

Please format your revised manuscript according to our Instructions to Authors https://academic.oup.com/braincomms/pages/General_Instructions
A summary of key points is attached to help with this. Please note that there can be substantial delays to publication if you don't resubmit in line with our requirements.

To revise your manuscript, click the link below or log into https://mc.manuscriptcentral.com/braincom and enter your Author Centre, where you will find your manuscript title listed under "Manuscripts with Decisions." Under "Actions," click on "Create a Revision." Your manuscript number will be appended to denote a revision.

*** PLEASE NOTE: This is a two-step process. After clicking on the link, you will be directed to a webpage to confirm. ***

https://mc.manuscriptcentral.com/braincom?URL_MASK=b8311346993d4742894a17b72ddeb07c

Please upload a revised manuscript with the changes you have made shown either with "track changes" or in coloured text.

When submitting your revised manuscript, you will be asked to respond to the reviewer(s) and editor's comments. Please be as specific as possible in your response to all of the comments.

The editorial team at Brain Communications also aims to increase transparency in scientific publishing and to demonstrate the value that peer review brings to the scientific process. Please therefore consider whether you are willing to have reviewer comments on your paper and your response published as supplementary data with the paper if it is accepted. This is entirely optional and will only be done if both the authors and reviewers agree. When submitting your revision you will have the opportunity to update the answer you entered previously.

IMPORTANT: Your original files are available to you when you upload your revised manuscript. Please delete any redundant files before completing the submission.

Because we are trying to facilitate timely publication of manuscripts submitted to Brain Communications, we would appreciate your revised manuscript as soon as possible. If it is not possible for you to submit your revision within 3 months, the option to submit a revision will expire, and we will consider your revised paper as a new submission. However, if you need a little more time, please get in touch and we can extend this deadline for you.

Please note that Brain Communications is a fully open access journal, and if your article is accepted for publication, an open access charge will be payable (this does not apply to letters and scientific commentaries). Details can be found in our Instructions for Authors: https://academic.oup.com/braincomms/pages/General_Instructions

Once again, thank you for submitting your manuscript to Brain Communications and I look forward to receiving your revision.

Best wishes

Dr. Manuela Marescotti
Scientific Editor, Brain Communications

Professor Tara Spires-Jones
Founding Editor, Brain Communications

Comments from the Scientific Editor:

- there is an issue with the ithenticate report (attached) related to the methods section. We cannot accept having portions of text completely copied and pasted from other articles. Please, edit this paragraph or replace the paragraph with the following wording: "the 'specified protocol' was performed accordingly to 'PAPER CITATION'.

- Fig3d: please report in the figure legend the relevant test statistic values alongside p values (for example F or t values for ANOVAs and t-tests).

- Fig1c: please, clarify if each data point represents a biological replicate, also, please since the number is <10 show the single data-points. Please note our policy on pseudoreplication, we do not accept analyses where individual cells are the experimental unit when there is likely biological variability between animals, culture replicates, etc. Please see instructions for authors for more details. For this type of data, we require either that a mean/median be taken per animal/culture replicate followed by statistical analyses or that more sophisticated analyses are performed such as mixed effects modelling which can account for random variables.

- Fig3b: please make sure to report in the figure legend, where missing, the test used and relevant test statistic values alongside p values (for example F or t values for ANOVAs and t-tests). Also, please, provide details of the experimental unit for each group analysed in these plots, and if the number is <10 show the single data-points. Please note: We do not accept analyses figure here individual measurements are the experimental unit when there is likely to be biological variability between animals, culture replicates, etc. For this type of data, we require either that a mean or median is taken per animal/culture replicate followed by statistical analyses, or that more sophisticated analyses are performed such as mixed effects modelling which can account for random effects.
